# Detection of cyber attacks in electric vehicle charging systems using a remaining useful life generative adversarial network

**DOI:** 10.1038/s41598-025-92895-9

**Published:** 2025-03-24

**Authors:** Hayriye Tanyıldız, Canan Batur Şahin, Özlem Batur Dinler, Hazem Migdady, Kashif Saleem, Aseel Smerat, Amir H. Gandomi, Laith Abualigah

**Affiliations:** 1 Faculty of Engineering and Natural Sciences, Malatya Turgut Ozal University, Malatya, 44210 Turkey; 2 CSMIS Department, Oman College of Management and Technology, 320 Barka, Oman; 3 Department of Computer Science & Engineering, College of Applied Studies & Community Service, King Saud University, Riyadh, 11362 Saudi Arabia; 4 Faculty of Educational Sciences, Al-Ahliyya Amman University, Amman, 19328 Jordan; 5 Centre for Research Impact & Outcome, Chitkara University Institute of Engineering and Technology, Chitkara University, Rajpura, 140401 Punjab India; 6 Faculty of Engineering and Information Technology, University of Technology Sydney, Ultimo, NSW 2007 Australia; 7 Computer Science Department, Al al-Bayt University, Mafraq, 25113 Jordan; 8 Computer Technologies Engineering, Mazaya University College, Nasiriyah, Iraq; 9 University Research and Innovation Center (EKIK), Obuda University, Budapest, Hungary; 10 Department of Computer Science, Khazar University, Baku, Azerbaijan

**Keywords:** Electric vehicle charging systems, Generative artificial intelligence, Remaining useful time, Cyber security, Energy science and technology, Engineering, Mathematics and computing

## Abstract

Cybersecurity attacks targeting electric vehicle supply equipment (EVSE) and the broader electric vehicle (EV) ecosystem have become an escalating concern with the increasing adoption of EVs and the growing connectivity of the infrastructure supporting them. The present research aims to contribute to continuing cybersecurity studies on electric vehicle charging stations. In line with this objective, this study proposes the remaining useful life (RUL) approach to demonstrate the potential impact of estimating the remaining time of a cyber attack on EVSE and what revolutionary changes it can bring to cyber security strategies using a generative adversarial network (GAN). By taking a proactive stance, the manuscript will increase security and reduce the economic and reputational losses associated with cyber incidents. Accurate RUL estimates present valuable information about the status of the EVSE infrastructure. Thus, informed decisions on maintenance and crew scheduling are taken. To test the technique’s effectiveness, we assess this approach on attack scenarios, including network and host attacks on the EV charger (Electric Vehicle Supply Equipment—EVSE) in idle and charging states. Furthermore, we assess the prediction results of different deep learning models, such as gated recurrent units (GRUs), long short-term memory (LSTM), recurrent neural networks (RNNs), convolution neural networks (CNNs), multi-layer perceptron (MLP), and dense layer integrated with generative adversarial networks (GANs), using mean absolute error (MAE), root mean square error (RMSE), mean squared error (MSE), and R-squared (R^2^). Afterward, we compare the error measurements with models, such as hybrid GAN-LSTM, GAN-GRU, GAN-RNN, GAN-CNN, GAN-MLP, and GAN-Dense Layer. The GAN-GRU model exhibits the highest accuracy with the lowest MAE (0.0281). On the contrary, the GAN-CNN model displays the best overall performance concerning error consistency and variance explained. According to the results, integrating GAN into these architectures improves predictive accuracy and the model’s ability to identify potential attacks in advance and decreases error rates.

## Introduction

The charging ecosystem of electric vehicles (EVs) represents a connected system paradigm at the smart grid’s center. It comprises a complex cyber-physical system with connected hardware and software elements and communication protocols. EVCS transfers power from the grid to EVs. The EVCS represents a self-contained and Internet-of-Things-enabled infrastructure operating on its proprietary firmware. The communication between public EVCS users and the charging management system is realized via the Internet. Users generally schedule charging sessions, establish the charging rate, start and terminate charging, and control the status of their EVs through the said services. The power infrastructure must be functional and connected to charge an EV. Since the EVCS is connected to the grid and obtains the required power, it considerably threatens the power supply’s safety and reliability. It is necessary to secure all data exchanged among the user application, EVCS, and EV to guarantee the ecosystem’s reliability and safety.

The subsequent wave in smart transportation aims to design renewable energy sources capable of fueling the automobile sector to ensure a shift toward Electric Vehicle Supply Equipment (EVSE). They have acquired more technological functions and characteristics in the last ten years, which increased their smartness and efficiency. At the same time, these innovations have subjected vehicles to new concerns and cybersecurity threats. Nevertheless, higher attention has yet to be paid to EV charging station cyber attacks on a broader industrial scale or at the level of government policies. There are common expectations that electric vehicle (EV) charging will open novel ways to increasingly influential cybersecurity risks for crucial energy and transportation infrastructures. However, a lot of cybersecurity risks and solutions are still unknown. Nowadays, vehicles are equipped with more software than the most advanced machines of the present day. The relevance of automotive software content and megatrends of electrification, connectivity, and autonomous driving have increased even more. The connectivity and complexity of software increase the vulnerability of cars to security threats.

The significance of energy management and transportation systems that utilize artificial intelligence in modern cities has increased since they developed the main urban infrastructures.

Threat modeling constitutes an acknowledged method for enumerating and characterizing potential threats and vulnerabilities that can cause a security incident if there is no suitable safeguard. The following analysis of the threat model guides and informs about countermeasures and prioritizes mitigations to prevent or decrease the influence of incidents. Threats are classified into one of the categories below, with the targeted features:

- Spoofing refers to masquerading as a legitimate user, process, or system element;
- Tampering indicates modifying or editing the legitimate information;
- Repudiation denotes denying or disowning a particular action that the system executes;
- Information disclosure represents data breach or unauthorized access to protected information;
- Denial of service refers to disrupting service for legitimate users;
- Elevation of privilege denotes acquiring higher privilege access to a system element by a user who has restricted authority^1^.

Generative Artificial Intelligence (GenAI) is a deep reinforcement learning method. The significance and influence of GenAI have increased in the world of empowering algorithms, technology, and models to produce highly realistic content autonomously. Due to the necessity of handling the evolving threat landscape caused by GenAI, we suggest leveraging the EVSE as the primary model for cyber defense. The current work is the first study to research in-depth cyber threats originating from the adversarial usage of GenAI within the context of EVSE. The present study explains how the RUL mechanism can be integrated into the generative adversarial training method to increase the security of Electric Vehicle Supply Equipment Systems (EVSE). The current study aims to contribute to continuing cybersecurity studies on Electric Vehicle Charging Stations by better understanding and applying methods for an effective cybersecurity strategy to estimate benign and attack scenarios based on the RUL strategy. The scenarios involve host and network attacks on the EV charger (Electric Vehicle Supply Equipment – EVSE) in idle and charging states. Network attacks comprise diverse Reconnaissance and Denial-of-Service (DoS) attacks, whereas host attacks involve backdoors and cryptojacking.

This paper provides an in-depth account of how the proposed hybrid model—built on deep reinforcement learning and artificial intelligence—operates, is implemented, and what its outcomes are. Additionally, it explores the model’s implications for shaping future cybersecurity strategies for Electric Vehicle Supply Equipment (EVSE) and outlines a potential roadmap for future research and development.

The novelties and contributions of the present study are summarized below:

- Estimating the time left to cyber attacks: The main aim of the manuscript is to develop data-based models that estimate the time left to a cyber attack and respond to cyber attacks more effectively and timely.
- Early warning and preventive actions: The work also aims to prevent possible attacks or reduce their potential effects by detecting the risks that may occur before cyber attacks.
- Behavior modeling and threat analysis: This involves analyzing attackers’ behavior and attack methods to predict future attack types.
- Ensuring real-time threat analysis and response capability for system security, analyzing aggressive behavior patterns specific to Electric Vehicle Supply Equipment Systems, and optimizing Remaining Useful Time (RUL) strategies by these models.
- Developing proactive cyber defense strategies: Developing proactive defense strategies that can respond to cyber attacks more quickly and effectively using prediction models.
- Review literature studies on security problems in EV charging, concentrating on cyber attack points and vulnerable communication protocols.

The remaining part of the manuscript is organized as follows: section “Literature survey” presents the literature survey. Section “Cyber attacks in EVSE” presents a background of recent advancements in EVCS. Section “Materials and methods” discusses the Materials and Methods. Section “The proposed model” addresses the suggested approach, while section “Experimental results” presents a conclusion and future studies.

## Literature survey

Comprehensive studies have been conducted on how the connectedness and computerization of modern vehicles represent cybersecurity risks to electric, connected, and autonomous vehicles. Recent research has presented examples of cyber attacks in electrical vehicle networks that can lead to severe failures like mechanical faults. This has made taking and developing cyber security measures a vital issue.

Buechler et al.^2^ introduced a GAN-based framework designed to capture the distribution of electric vehicle charging sessions while also disentangling underlying representations. Their study revealed that this approach can autonomously parameterize both temporal and power patterns from unlabeled data, subsequently generating synthetic data that adheres to these parameters. Additionally, by comparing its performance with that of Gaussian Mixture Models (GMMs), they demonstrated that their framework more effectively modeled the charging session distributions and their temporal dynamics. Çolak et al.^3^ reviewed the literature on EV technologies, concentrating on diverse aspects of EV charging systems, including charging optimization standards and techniques. This review aimed to determine critical factors capable of minimizing charging times, improving energy efficiency, and extending the lifespan of a battery, therefore facilitating the adoption of EVs on a broader scale. Hasib et al.^4^ comprehensively reviewed the current battery datasets, remaining useful life (RUL) prediction approaches, and advanced battery management techniques. The review investigated various datasets available for battery research, advanced battery management strategies, and RUL prediction methods. Also, mainly focused on data sets, battery functioning and management, and RUL prediction approaches.

Linru et al.^5^ considered the problem of EV security. The safety issue was discussed from the perspective of the charging process and the charging equipment operation. The authors analyzed the statistics of charging failure cases. They took into account the failure caused by the charging equipment and researched the malfunctions of the EV itself. By analyzing equipment failure cases and their operation, the authors found a car’s safety index and offered solutions to charging problems. A systematic approach and a comprehensive solution allow for controlling the charging process. Some countermeasures help evaluate an electric car’s safety index, identify non-faults, and create a complete system of charging safety standards. Wang and Wang^6^ studied electrical safety and prevention in electric charging of EVs. The work’s primary goals were to research degradation, protection failures, cyber attack challenges, and mismatch between supply and demand. Jeong and Choi^7^ presented a cyber attack aiming at manipulating EV user data against the energy management system (EMS) of an EVCS, which could result in an incorrect calculation of the electricity costs incurred by the EVCS. The work should have considered online cyber attack detection and ignored the existing secure protocol. Akbarian et al.^8^ addressed this issue through a novel two-step framework reinforced by a machine learning (ML)-based attack detector. Xu et al.^9^ depicted and analyzed the principle and security threats of the charging protocol between electric vehicles and charging piles. The researchers also introduced a fuzzy-based detection method. Ultimately, they presented the authentication mechanism, targeting the charging protocol’s security problems. Shezaf Ofer^10^ explained the possible threats of charging piles in five dimensions: short-range communications, physical access, the Internet of Things, encryption, and the human factor.

The authors in^11^ highlighted the potential for eavesdropping on the communication between EVCS and charging station operators. Furthermore, they demonstrated that Disruption could occur by deleting or replaying specific messages during communication. The study^12^ identified the feasibility of a false data injection attack (FDIA) targeting the data market for EVCSs and proposed a defense mechanism to counter such attacks. Although the highly cited references emphasize the vulnerability of EVCSs to FDIAs, they ignore the potential of machine learning algorithms (MLAs) as a highly effective mitigation technology. Acharya^13^ took advantage of ML techniques to detect abnormalities in the charging rate. Ahalawat et al.^14^ comprehensively addressed security threats in EV charging systems. The researchers also examined the charging station system’s architecture and the protocols between charging stations and electric vehicles. Basnet et al.^15^ introduced diverse attack types on electric vehicle charging systems. The said work addressed FDI and syn-flood DDoS attacks on 5G-enabled supervisory control and data acquisition (SCADA) systems and remote control, battery energy storage (BES) controller, Electric Vehicles Charging Station (EVCS) controller and EV controller, and photovoltaic solar photovoltaics (PV) controller. The researchers employed the deep learning-based approach to detect attacks in the EV system. They developed and tested an LSTM-based stack-based local IDS based only on the suggested Electric Vehicle Supply Equipment (EVSE) based on electrical fingerprinting.

Babu et al.^16^ addressed a novel technology trend of dynamic electric vehicle charging, altering the transport infrastructure paradigm and considerably influencing people’s daily lives. However, various common threats, such as reuse, fraud, man-in-the-middle attacks, etc., threaten the interaction between parties that play a role in dynamic charging systems. The above manuscript also addressed the benefits and privacy problems of connecting electric vehicles to dynamic charging stations. A classification system of security protocols for communication between dynamic charging stations and electric vehicles was also established, involving lightness, authentication, secure billing, confidentiality, and issuance of payment means. Hamdare et al.^17^ analyzed the cybersecurity risks of the EVCS network. First, the researchers described recent EV adaptation trends, recent developments in the EVCS network, and charging use cases as a background of the study area. Second, the study explored cybersecurity issues within EV charging stations by examining vulnerabilities in both infrastructure and communication protocols that could be exploited by cyber-attacks. Third, the researchers confirmed these risks through real-time, data-focused analysis of EV charging sessions. Moreover, the study highlighted several unresolved research challenges in EV cybersecurity, offering new insights for both practitioners and domain experts.

This article has provided an in-depth overview and assessment of the cybersecurity vulnerabilities concerning the EV charging systems, outlining the gaps as well as the requisite measures that need to be implemented for EVs operating within the interconnected and automated framework. It was stressed that some of the problems such as the EV charging infrastructures being tampered with: communication breakdowns and evm data being changed around among many others are issues that must be resolved. To tackle these risks, measures have been developed such as; security protocols, data mining techniques, detection algorithms, and many more. But there is a clear need for further work on; the creation of machine learning algorithms aimed at identifying anomalies, the real time detection of cyber attacks, the introduction of unified security standards for all the networks of EV charging stations. This body of research suggests that in order to guarantee the routine use of cyber engineered EV’s, enhancing control measures and technological standards is a long term goal which would not just geographically limit the vulnerabilities but would reshape the cybersecurity landscape for the better.

## Cyber attacks in EVSE

**Fig. 1:**
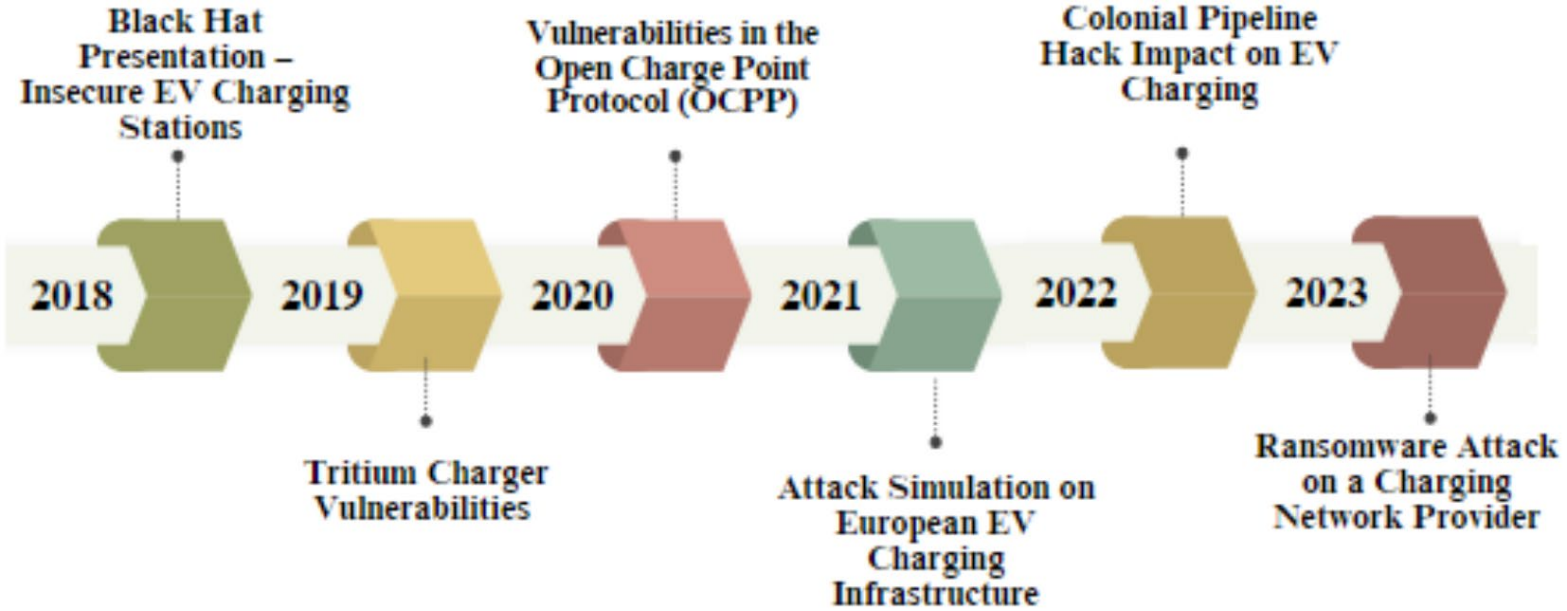
Timeline and history of EVSE cybersecurity attacks.

A cyber attack is a deliberate attempt by individuals, groups, or organizations to breach the security of computer systems, networks, or devices to steal, alter, damage, or disrupt information or operations. A cyberattack on EVSE refers to any malicious activity that targets digital systems, communication networks, or software controlling or operating EV charging stations. Figure 1 displays the historical background of cyberattacks targeting to disrupt services, steal sensitive data, manipulate the charging process, or acquire unauthorized access to critical infrastructure.

**The 2018 Black Hat Presentation—Insecure EV Charging Stations**: Researchers demonstrated vulnerabilities in several popular EV charging stations at the Black Hat cybersecurity conference. They highlighted how weak security protocols could allow attackers to disable or shut down the charging station, manipulate power settings, launch denial-of-service (DoS) attacks, and steal user data or billing information. These weaknesses could lead to large-scale disruptions if an attacker compromised multiple charging stations. The 2019s-**Tritium Charger Vulnerabilities**: Researchers discovered security flaws in Tritium’s Veefil-RT chargers. Using these vulnerabilities, potential attackers could access the charging station’s management system, modify software configurations, and collect private data, such as users’ billing and personal information. Tritium quickly issued a software update to address these security flaws (patch). **The 2020s-Vulnerabilities in the Open Charge Point Protocol (OCPP): The** OCPP represents a widely adopted communication protocol between central management systems and charging stations. Researchers discovered numerous security weaknesses, including unencrypted communications, increasing vulnerability to interception, and insecure authentication mechanisms.

Using the weaknesses, attackers could steal user data, manipulate the charging process, or disrupt power grids. **The 2021s-Attack Simulation on European EV Charging Infrastructure**: A team of European cybersecurity experts simulated an attack on a network of EV charging stations and showed how attackers could control multiple stations simultaneously and disrupt grid operations by manipulating the charging load. Large-scale manipulation could cause power grid instability or overload, influencing EV users and broader electrical services. **The 2022s-Colonial Pipeline Hack Impact on EV Charging**: Although the Colonial Pipeline cyberattack’s primary target was the fuel supply system, the incident raised concerns about the EV charging infrastructure’s indirect vulnerabilities. Since conventional fuel supplies were compromised, demand for EV charging increased dramatically. This increase emphasized potential risks in case of future cyberattacks that are similar to those that are targeting EV charging systems. The importance of integrating robust cybersecurity measures into EVSE was researched. **The 2023s-Ransomware Attack on a Charging Network Provider**: A ransomware attack targeted a significant charging network provider, temporarily turning off a few charging stations across the U.S. and Europe. The attackers demanded cryptocurrency in exchange for restoring service. Although the affected company restored operations without paying the ransom, the incident emphasized the increasing cyber risk for EVSE providers.

### Cyber attack scenarios targeting electric vehicles, electric vehicle supply equipment (EVSE), and charging infrastructure

As electric vehicles (EVs) are adopted increasingly, the complexity and interconnectivity of the charging infrastructure also increase, exposing these systems to cybersecurity vulnerabilities. Electric Vehicle Supply Equipment (EVSE) and the broader charging network form critical links between EVs, power grids, and communication networks, making them attractive targets for cyber attacks. Below, we describe various potential cyber attack scenarios that target these components, highlighting the associated risks and implications for EV infrastructure security. Table 1 summarizes these attacks’ details.

**Table 1:** The details of the common cyber attack scenarios in EVS^18,19^.

Types of cyber attacks	Description	Impact
Denial of Service (DoS) Attack	In a DoS attack, attackers overload a system or network with traffic, making it unavailable to legitimate users	Disruption of charging services, preventing users from charging their vehicles
		Potential grid instability if coordinated with other disruptions
		In-vehicle systems become unresponsive, affecting navigation or autonomous features
Man-in-the-Middle (MitM) Attack	In a MitM attack, attackers intercept and possibly change communication between two parties without their knowledge	Theft of sensitive data (e.g., payment information, user credentials)
		Unauthorized control over vehicle features (e.g., remote unlocking or starting)
		Manipulation of charging data, causing inaccurate billing or energy manipulation
Ransomware	Ransomware locks the system or encrypts data, demanding payment for access or functionality restoration	Disabling of critical vehicle functions such as driving or charging
		Possible widespread impact if targeted against charging infrastructure, causing service outages
		Data loss or Theft if the attack includes exfiltration prior to encryption
Remote Vehicle Hijacking	Hackers gain unauthorized control over a vehicle’s systems through software or wireless connection vulnerabilities	Attackers take control of critical vehicle operations (e.g., steering, acceleration, braking)
		Risk to passengers and other road users if vehicles are compromised in motion
		Potential for mass Disruption if autonomous vehicles are targeted at scale
Firmware and Software Exploits	Attackers exploit vulnerabilities in the vehicle’s firmware or software to inject malicious code or compromise system integrity	Introduction of backdoors for future attacks
		Manipulation of vehicle performance (e.g., reducing battery efficiency or sabotaging key components)
		Unauthorized access to user data or sensitive vehicle information
Data Theft and Privacy Attacks	The attacks aim to steal or misuse sensitive data transmitted between vehicles and external systems (e.g., user information, location, driving behavior)	Theft of personal or financial information stored in the vehicle or cloud
		Tracking of vehicle movements, leading to privacy violations
		Sale of stolen data on the black market
V2X (Vehicle-to-Everything) Communication Attacks	V2X communication allows data exchange among vehicles, road infrastructure, and networks, making them a potential target for interference or spoofing attacks	False data can cause accidents by misleading vehicles about road conditions or traffic
		Compromising traffic lights or signs can disrupt traffic flow and safety
		Interference with fleet management systems can cause logistical challenges for commercial vehicles
Battery Management System (BMS) Attacks	These attacks focus on manipulating the EV’s BMS to cause battery inefficiencies, premature wear, or potential safety hazards	Overcharging or undercharging the battery, reducing its lifespan
		Creating overheating risks that can lead to fires or explosions
		Disruption of grid power by manipulating the charging load on a large scale
Supply Chain Attacks	Attackers infiltrate the EV supply chain by compromising hardware or software	Introduction of vulnerabilities or malware in the vehicle’s software or hardware
		Broad influence across numerous models or manufacturers
		Long-term vulnerabilities that are challenging to detect and patch
Charge Point Exploits	Attackers exploit public or private charging station vulnerabilities to steal payment information, alter charging rates, or deny service	Financial loss by stealing or manipulating billing information
		Disruption of charging services for multiple users
		Potential exploitation of charging station vulnerabilities to attack connected EVs
Vehicle Identity Spoofing	Attackers spoof a vehicle’s identity by falsifying its unique identifiers (e.g., the VIN number or registration data)	Fraudulent usage of charging services or access to restricted areas (e.g., toll roads, parking lots)
		Misleading authorities concerning the location or owner of the vehicle
		Possible criminal activities using a falsified vehicle identity
Brute Force Attacks on Access Systems	Attackers use brute force techniques to break into the vehicle’s access control systems (e.g., keyless entry or mobile application controls)	Stealing the vehicle or unauthorized access to it
		Tampering with vehicle settings (e.g., location tracking, remote start)
		Compromise of associated data, such as GPS history or saved addresses

### Solutions to cyber attack scenarios targeting electric vehicles, electric vehicle supply equipment (EVSE), and charging infrastructure

Developing practical solutions to mitigate potential cyber attack threats becomes more crucial when integrating electric vehicles (EVs) and their associated infrastructure, such as electric vehicle supply equipment (EVSE), into modern transportation and energy systems. The solutions address communication, hardware, and software vulnerabilities that attackers can exploit. Below, we present several key strategies to secure EVs, EVSEs, and charging infrastructure against cyber attacks.

1. End-to-End Encryption: To decrease the risk of Man-in-the-Middle (MitM) attacks, it is essential to encrypt communications between EVs, EVSE, and backend systems end-to-end. When robust encryption protocols are implemented, unauthorized third parties cannot intercept or alter any data transmitted over the network—whether regarding charging, payments, or vehicle diagnostics. Furthermore, secure communication standards, e.g., Transport Layer Security (TLS), can be applied to protect sensitive information and maintain data integrity during transmission.
2. Multi-Factor Authentication (MFA): It is possible to prevent unauthorized access to EVSE systems by introducing multi-factor authentication (MFA) for user and system access. MFA requires numerous verification methods (e.g., passwords, tokens, biometrics) before granting access, significantly reducing the likelihood of attackers gaining control of critical systems. It is possible to minimize the risk of cyber attacks by securing user authentication and ensuring that only verified users can access charging systems.
3. Intrusion Detection and Prevention Systems (IDPSs): Threats can be identified and neutralized in real-time by utilizing advanced Intrusion Detection and Prevention Systems (IDPSs) within EV charging networks. The systems mentioned above monitor network traffic and charging station activities for abnormal behavior, e.g., excessive requests (indicative of Denial of Service (DoS) attacks), suspicious login attempts, or unusual communication patterns between the EV and the charger. When a potential threat is detected, the IDPS can alert administrators or automatically take action to block the attack.
4. Regular Firmware and Software Updates: It is crucial to update firmware and software regularly to ensure defense against cyber attacks. Manufacturers of EVs, EVSEs, and associated systems must perform security updates regularly to patch known vulnerabilities. Automatic or remote updates can ensure the security of all charging infrastructure components, from the vehicle’s onboard software to the charging station’s operating system, against evolving threats.
5. Network Segmentation: Segmenting the network connecting EVSE, vehicles, and backend systems can limit the spread of cyber attacks. When critical systems are isolated from less secure ones, an attack compromising one part of the network will not necessarily impact the whole system. For example, an attack on the payment interface will not disrupt charging operations or compromise vehicle data if payment processing systems are separated from charging management systems.
6. Blockchain for Secure Transactions: Integrating blockchain technology into the EV charging infrastructure can enhance the security of financial transactions. The decentralized and immutable nature of the blockchain ensures secure, transparent, and tamper-proof payment processing, protecting charging fees and other monetary exchanges between the vehicle, EVSE, and energy providers against tampering or fraud.
7. Secure Vehicle-to-Grid (V2G) Communication Protocols: Vehicle-to-Grid (V2G) communication provides bidirectional energy flow between EVs and the energy grid. However, it also creates potential vulnerabilities. It is necessary to implement secure V2G protocols to protect this communication’s integrity. It is possible to prevent malicious actors from utilizing this connection to disrupt energy flow, cause grid instability, or manipulate vehicle charging states using cryptographic methods and authentication protocols specific to V2G technology.
8. Redundancy and Resilience Planning: Developing robust resilience strategies, including redundancy within the EVSE network, can ensure continued operation during an attack. This may involve backup communication systems, failover charging stations, or contingency plans to reroute energy in case of a DoS attack or other Disruption. An attack’s impact can be mitigated through resilient infrastructure design, and service availability to EV users can be provided even during security incidents.
9. Artificial Intelligence-Based Threat Detection: Applying AI-driven solutions to monitor and predict potential security breaches represents a forward-looking approach toward securing EVSE networks. It is possible to train machine learning models on network traffic data to detect anomalies and preemptively block attacks before the damage. These AI systems can evolve with emerging threats, increasing their accuracy over time and ensuring that even sophisticated attacks are caught in the early period.
10. Security by Design: Finally, cybersecurity is integrated into every product development phase by adopting a “security by design” approach toward developing EVs, EVSE, and associated infrastructure. Security measures must be embedded within the core design, from the hardware level (e.g., secure processors) to the software and network protocols. The surface area available for attacks can be minimized, and the need for subsequent reactive patching can be decreased by adopting the proactive approach mentioned above.

## Materials and methods

### Deep learning classifiers

Deep Learning (DL), which represents a subset of machine learning (ML), replicates the way the human brain interprets and processes information using neural networks, intense neural networks (DNNs) that consist of multiple layers. These architectures are good at automatically learning and extracting hierarchical features from raw data. This makes them especially effective in dealing with complex and large datasets. DL models have achieved substantial success across various applications, such as image and speech recognition and natural language processing, which shows their versatility and effectiveness in solving complex problems.

#### Recurrent neural networks (RNNs)

**Fig. 2:**
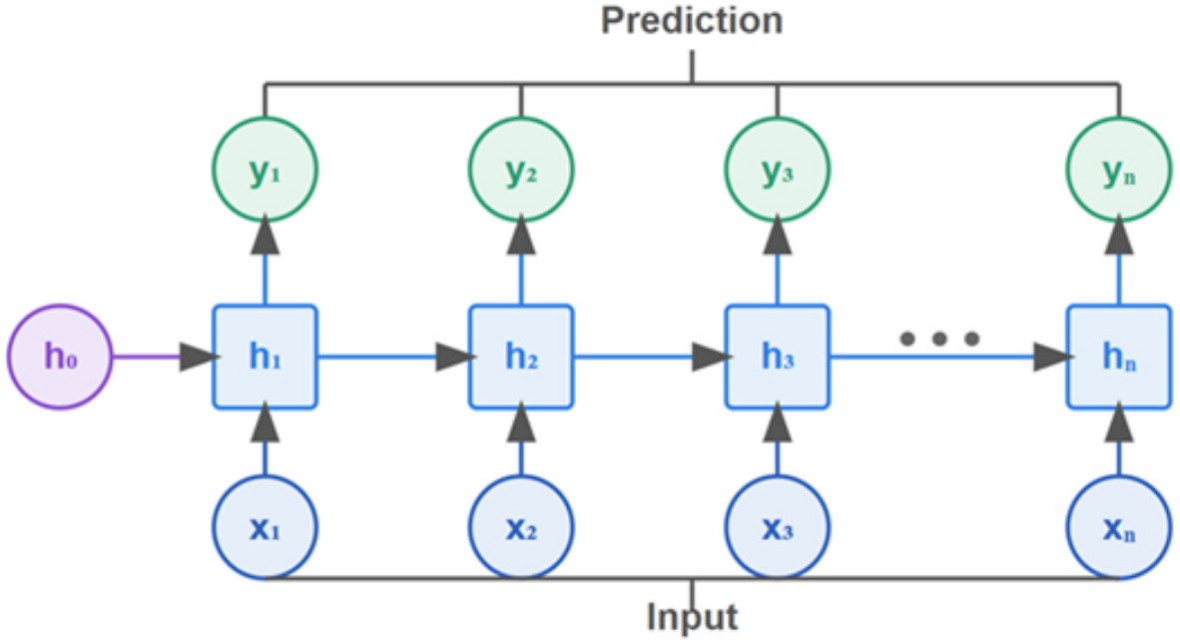
Recurrent neural networks^20^.

Recurrent Neural Networks (RNNs) are a category of deep learning models that incorporate an internal memory mechanism, allowing them to capture dependencies across sequences. Unlike traditional neural networks, which treat each input as an isolated unit, RNNs take the order of inputs into account, making them well-suited for sequential tasks. They operate by looping through each element in a sequence, where the output at each step is influenced by both the current input and the previous computations. A simple recurrent neural network is shown in Fig. 2, in which the internal memory (${h_t}$) is calculated using Eq. (1):

1 $${h_t}=tanh\left( {{X_t}U+{h_{t - 1}}W+b} \right)~~$$

Where$~{X_t}$ denotes the sequence input at time *t*, ${h_{t - 1}}$ refers to the cache’s past information, and *U*, *W*, and *b* represent input weight, caches previous information weight, and bias, respectively, for an RNN current cell state.

#### Long short-term memory (LSTM)

**Fig. 3:**
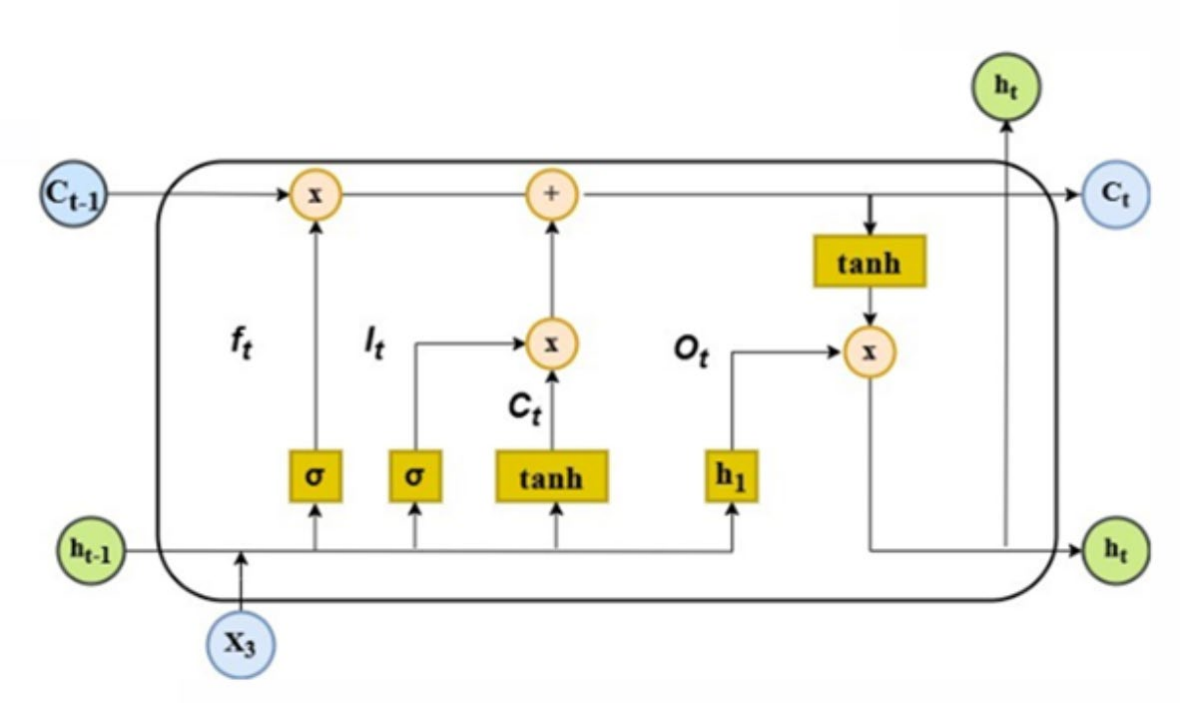
LSTM recurrent neural networks^22^.

A Long Short-Term Memory (LSTM) network is a type of recurrent neural network (RNN) that excels at capturing long-term dependencies. Unlike a feedforward network, which simply converts an input vector to an output vector, the LSTM can selectively store and accumulate information over time. The system in Fig. 3 presents the computation of the cell’s concealed state. The system has three gates: input, forget, and output. In Eq. (2), the input gate manages the stream of the novel information in the memory unit ${i_t}$. For all equations, ${x_t}$ refers to input data,$~{h_{t - 1}}$ denotes the previous time step’s concealed state, and ${W^i}$ and ${U^i}$ refer to input gate weights, with ${b^i}$ being the input gate bias^21^.

2 $${i_t}=\sigma \left( {{x_t}{U^i}+{h_{t - 1}}{W^i}+{b^i}} \right)$$

The forgetting gate ${f_t}$ employs a sigmoid activation to decide which elements of the previous hidden state ${h_{t - 1}}$ should be discarded. Eq. (3) illustrates how the actual input ${f_{t~}}$ for the forgetting gate is computed by combining the gate’s weight matrices ${W^f}$, $~{U^f}$, with its bias ${b^f}.$

3 $${f_t}=\sigma \left( {{x_t}{U^f}+{h_{t - 1}}{W^f}+{b^f}} \right)$$

Equation (4) illustrates how the output gate ${o_t}$ governs the flow of information necessary for activating the memory unit’s output and ${W^o}$ and $~{U^o}$ represent the weights associated with the output gate, while ${b^o}$ denotes its bias.

4 $${o_t}=\sigma \left( {{x_t}{U^o}+{h_{t - 1}}{W^o}+{b^o}} \right)$$

Equation (5) demonstrates that the input gate ${i_t}$ interacts with the cell’s previous state ${C_{t - 1}}.$ ${C_t}$ explains how the current cell state, which is updated by integrating the effect of the forgetting gate ${f_t}$ and $~{\tilde {C}_t},$ represents a potential new state at time step $\left( t \right),~$and it is represented in Eq. (6).

5 $${C_t}=\sigma \left( {{i_t}*{{\tilde {C}}_t}+{f_t}*{C_{t - 1}}} \right)$$

6 $${\tilde {C}_t}=tanh\left( {{h_{t - 1}}{W^c}+{x_t}{U^c}+{b^c}} \right)$$

${W^c}$,$~{U^c},$ and ${b^c}~$denote the candidate’s weights and bias for the current unit state, respectively, and the symbol * indicates the Hadamard product.

Equation (7) computes the hidden state ${h_t}$ . In this step, the output gate ${o_t}$ is applied alongside the *tanh* activation function to the current cell state ${C_t}$. This updated state is then forwarded to the next memory unit.

7 $${h_t}=tanh\left( {{C_t}} \right)*{o_t}$$

The LSTM model controls the input information flow, which ensures that the cell learns dependencies longer. The parameters of the diverse logic gates weight the gradient computation selectively, as displayed in Eqs. (5) and (7). This decreases the recurrent impact of extreme values, stabilizing the optimization process. Furthermore, it ensures that the model retains information over longer steps.

#### Gated recurrent unit (GRU)

**Fig. 4:**
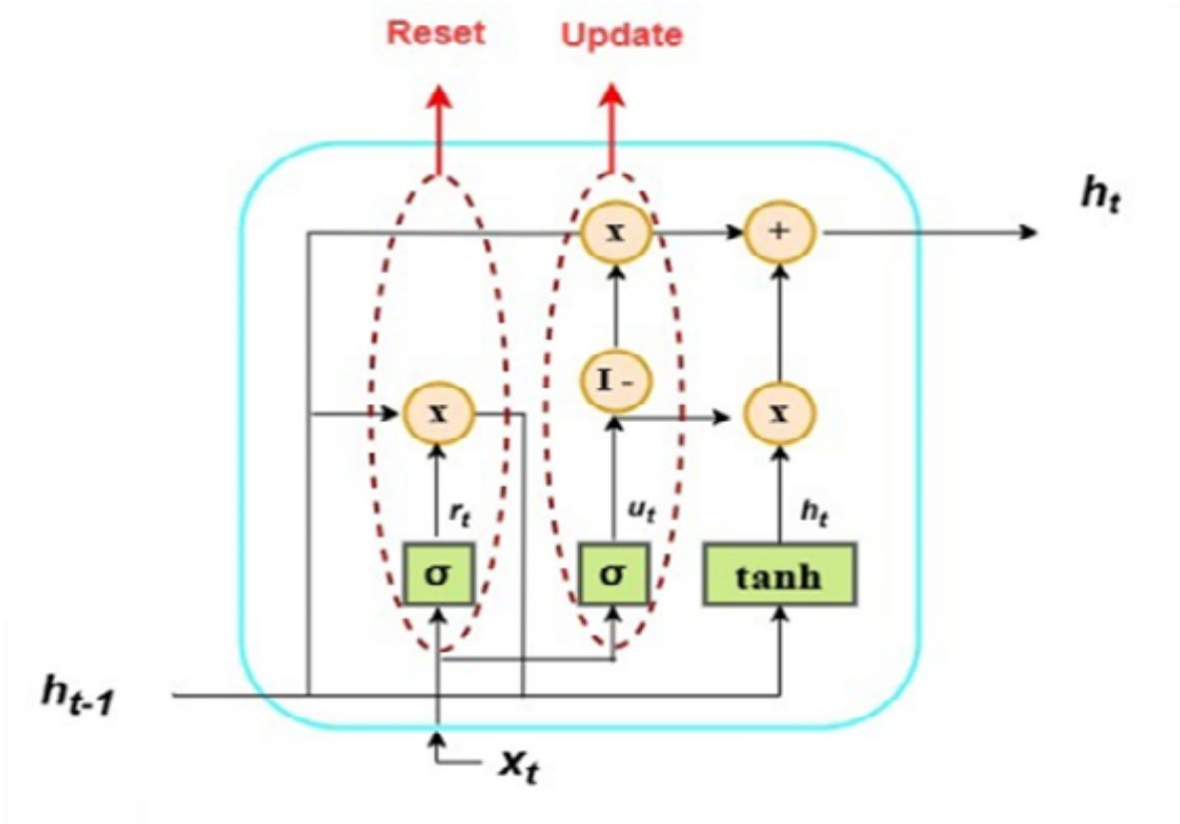
GRU recurrent neural networks^23^.

The Gated Recurrent Unit (GRU) represents the recurrent neural network (RNN) structure type. The GRU helps RNNs learn long-string dependencies more effectively. The GRU model is similar to the LSTM. However, it has only two gates. The system in Fig. 4 represents the computation of the cell’s concealed state, where it has reset and update gates denoted by $r$ and $z,$ respectively. $x$ denotes input, ${h_{t - 1}}$ represents the previous step’s output, and ${h_t}$ refers to the output. As shown in the LSTM model, $W$ and $U$ denote weights, and the symbol $b$ represents the bias for all equations. Equation (8) depicts the output of the reset gate. This gate exhibits traits similar to the LSTM cell’s forgetting gate, and it produces a reset vector *r* that identifies which information from earlier hidden states will be removed.

8 $${r_t}=\sigma \left( {{h_{t - 1}}{U^r}+{x_t}{W^r}+{b^r}} \right)$$

The update gate determines how much of the previous information should be carried forward. This capability is crucial because it allows the model to preserve all relevant past data, effectively mitigating the vanishing gradient problem. Equation (9) shows the update gate’s output equation. 

9 $${z_t}=\sigma \left( {{h_{t - 1}}{U^z}+{x_t}{W^z}+{b^z}} \right)$$

Equation (10) sets the implied output but not the target output. The reset gate’s output will influence the implied output at the point in question.

10 $${\tilde {h}_t}=tanh\left( {{x_t}W+{r_t}*{h_{t - 1}}U} \right)$$

Equation (11) determines the target output set from the last and implied outputs. If the update gate’s value is close to zero, feature data from the previous step are required. The symbol * shows the Hadamard product.

11 $${h_t}=\left( {1 - {z_{t~}}} \right)*{h_{t - 1}}+{z_t}*{\tilde {h}_t}$$

#### Convolutional neural networks (CNNs)

**Fig. 5:**
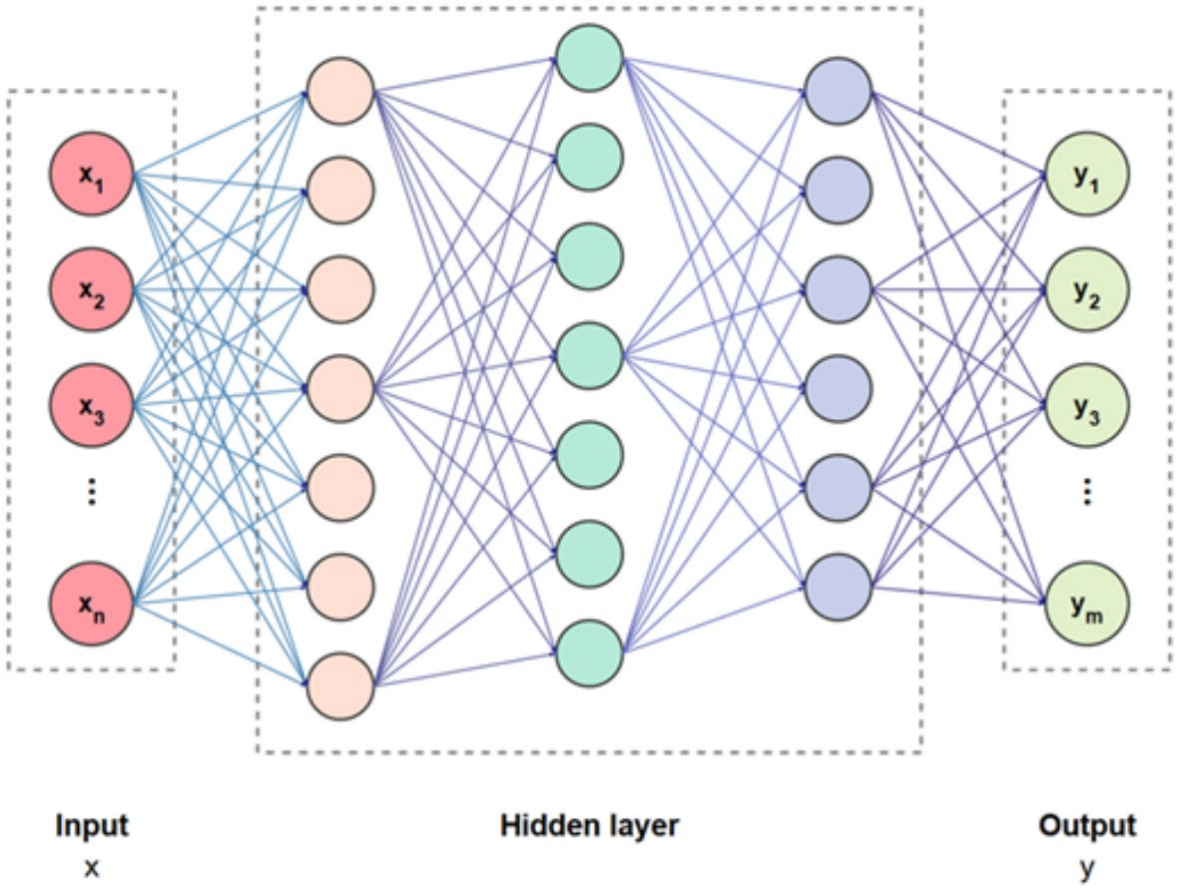
Framework of CNN model^24^.

CNNs apply the basic concept of the Neural Network (NN) algorithm with more layers. Figure 5 displays a CNN comprising input/output layers, hidden layers, and a completely connected network. In the hidden convolution layer, the fundamental operation in CNNs is the convolution operation. With an input feature map I and a convolution kernel K, the convolution operation is described in the following way:

12 $$\left( {I*K} \right)\left( {x,y} \right) = \mathop \sum \limits_{i} \mathop \sum \limits_{j} I\left( {x + i,y + j) \times K\left( {i,j} \right)} \right)$$

where $\left( {x,y} \right)$ denotes the spatial position in the output feature map and $I\left( {x+i,y+j} \right)$ refers to the input at spatial position $\left( {x+i,y+j} \right)$. The convolution operation calculates the input values’ weighted sum by sliding the kernel over the input feature map. An activation function is implemented element-wise to introduce nonlinearity following the convolution operation. ReLU and sigmoid are among the frequently used activation functions. The operation above is expressed in the following way in mathematical terms:

13 $$A\left( {x,y} \right)=f\left( {I*K} \right)\left( {x,y} \right)$$

Pooling layers reduce the feature map’s spatial dimensions. Max pooling represents the most frequently used pooling operation, in which the highest value in a local region is retained. It is possible to define the max-pooling operation as shown below:

14 $$P\left( {x,y} \right)=\mathop {\hbox{max} }\limits_{{i,j}} A\left( {x.s+i,y.s+j} \right)~$$

Where $P\left( {x,y} \right)$ indicates the pooling layer’s output, $s$ denotes the stride, and $A\left( {x.s+i,y.s+j} \right)~$shows the input to the pooling layer.

In the final stage of a CNN, one or more fully connected layers are employed for tasks such as classification. These layers flatten the output feature maps and establish connections by linking every neuron in the flattened vector to all the neurons in the preceding layer. The complete operation of a CNN—from the convolution processes to the fully connected layers—can be mathematically represented as follows:

15 $$Y=f\left( {W*X+B} \right)$$

Where $Y$ refers to the output, $W$ denotes the weights (learned during training), $X$ represents the input data, $B$ represents the bias term, $*$refers to the convolution operation, and $f$ represents the activation function.

#### Multi-layer perceptron (MLP)

An MLP represents a perceptron learning rule based on a computing model in artificial neural networks. It is a supervised learning network comprising input, hidden, and output layers. A hidden layer occurs between an input and output layer. In this neural network architecture, the input layer connects to the hidden layer, and the hidden layer, in turn, connects to the output layer via weighted links. Initially, input data is fed into the network, and the net input is computed as the sum of these inputs multiplied by their respective weights. This aggregated value is then passed through a sigmoid activation function to generate the output. The network repeats this process until a stopping criterion—typically a minimal error threshold—is reached. Performance is subsequently evaluated using test data. Essentially, the network operates in two main stages: first, it computes the output for each layer; and second, if the actual output does not match the expected result, it updates the weights to reduce the error. The hyperbolic sigmoid activation function is executed on the net input to calculate the output. The neural network’s output is presented below:

16 $$Y=f\left( {\mathop \sum \limits_{{j=1}}^{m} ZjWj} \right)$$

Where $Zj$ refers to the hidden layer’s output, $Wj$ represents the weight between the hidden and output layer, $m$ denotes the number of hidden neurons, and $f$ shows the activation function.

#### Dense layer

A dense layer (also referred to as a fully connected layer) is a core building block of many neural networks, especially in feedforward and deep learning architectures. In a dense layer, every neuron (or node) is connected to every neuron in the previous and subsequent layers. Such dense connectivity ensures the model learns complex patterns and relationships within the input data.

In the dense layer, the input features are multiplied by a weight matrix and summed with a bias term, after which an activation function is executed. This operation can be expressed in mathematical terms as follows:

17 $$Y=f\left( {Wx+b} \right)$$

where $W$ denotes the weight matrix containing the layer’s learnable parameters, $x$represents the input vector, $b$ is the bias vector, which helps to shift the activation function, and $f$ represents the activation function applied element-wise to the output of the linear transformation.

Dense layers are typically utilized later in a neural network, particularly in tasks requiring classification or regression. They map the high-level representations learned by previous layers into output predictions. Depending on the task’s nature, ReLU (Rectified Linear Unit), sigmoid, and softmax are listed among the standard activation functions utilized in dense layers.

From a specific perspective, dense layers ensure that neural networks learn complex patterns in data using optimization techniques, such as backpropagation. Because of the full connectivity between neurons, they are computationally intensive. Nevertheless, they constitute potent tools for shallow and deep neural networks. Dense layers are essential for final decision-making processes in tasks, including image classification, natural language processing, and time-series forecasting.

Dense layers’ versatility and simplicity make them indispensable components in neural networks. They contribute significantly to model performance when used in conjunction with other layer types (e.g., convolutional or recurrent layers) in more complex architectures.

#### Generative adversarial networks

Ian Goodfellow presented generative adversarial networks, or GANs, for the first time in 2014. The minimax two-person zero-sum game, where one player gains profit only when the other obtains an equal loss, forms the basis of the GAN. The generator and the discriminator constitute the two players in the GAN. The goal of the generator is to deceive the discriminator. On the other hand, the goal of the discriminator is to determine whether a sample is from an actual distribution. A probability that the input sample is an actual sample represents the output of the discriminator. The Eq. (18) describes the GAN architecture’s objective function:

18 $$\arg \mathop {\hbox{min} }\limits_{G} \mathop {\hbox{max} }\limits_{D} V\left( {D,~G} \right)=~{{\mathbb{E}}_{\left\{ {x\sim {p_{data\left( x \right)}}} \right\}\left[ {\log D\left( x \right)} \right]}}+~{{\mathbb{E}}_{\left\{ {z\sim {p_{z\left( z \right)}}} \right\}\left[ {\log \left( {1~ - ~D\left( {G\left( z \right)} \right)} \right)} \right]}}$$

where $D\left( x \right)$ denotes the discriminator function, the function leads to a probability that the input vector denoted by $x$ is from the training dataset. By taking $x$ as input, $D\left( x \right)$ generates a value in the range of 0–1. Likewise, $G\left( z \right)$is is known as the generator function causing a matrix, the dimension of which is identical to that of $x$ depending on the $z$ (noise vector). From the training dataset, ${P_{data}}\left( x \right)$ denotes the samples’ probability distribution.$~{P_z}\left( z \right)$ refers to the probability distribution of samples acquired from the noise generator. $E\left( \cdot \right)$ denotes the expectation function obtained from the log-loss function as a positive class. Equation (19) depicts the log-loss function:

19 $$E\left( {\left( {\left. p \right|~y} \right)} \right.~ = - ~1/N~\sum \_\left\{ {i = 1} \right\} \wedge \left\{ N \right\}~\left( {~y\_i~\log p\_i~ + ~\left( {1~ - ~y\_i} \right)~\log \left( {1~ - ~p\_i} \right)~} \right)$$

**Fig. 6:**
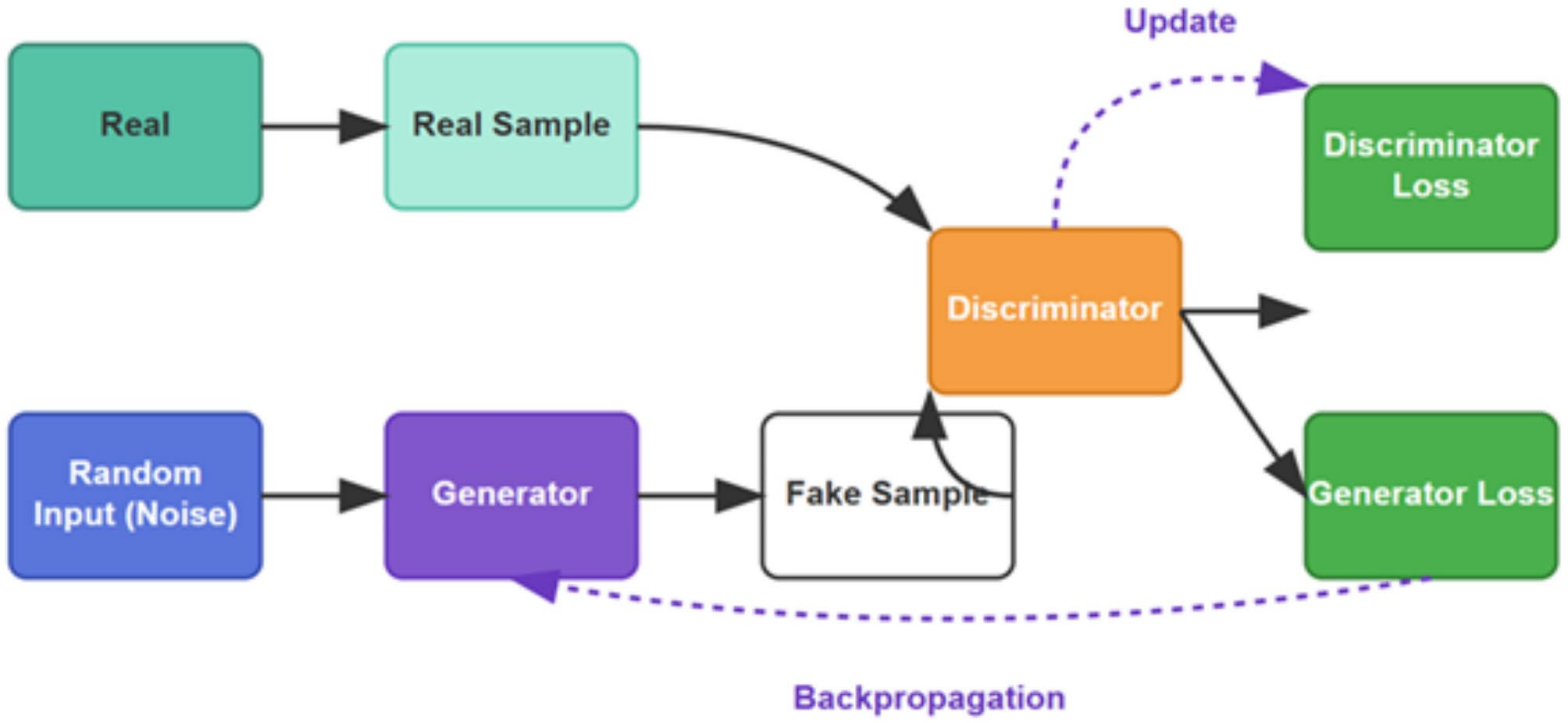
Generative adversarial networks^25^.

Where ${y_i}$ refers to the actual data, and ${p_i}$ refers to the estimation. The log function is employed in case 0 or 1 is expected as a response from the model. Figure 6 displays the GAN’s architecture.

## The proposed model

**Fig. 7:**
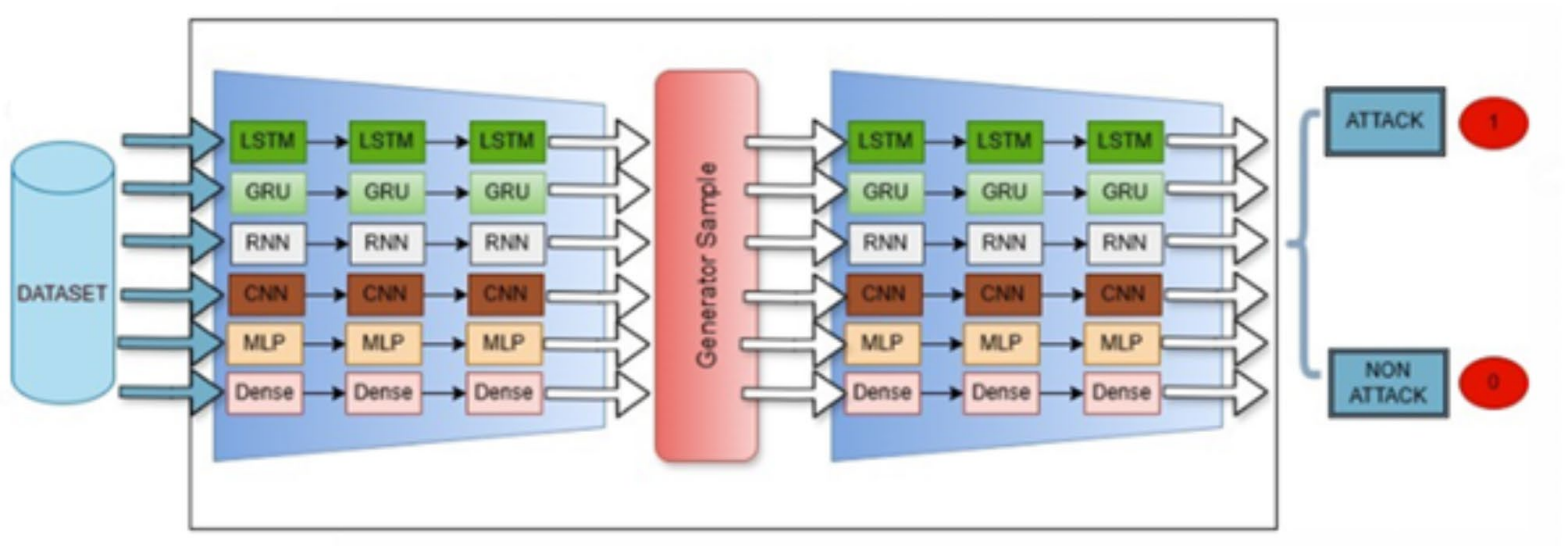
The structure of the proposed GAN-DL model.

This study aims to establish a pre-warning system by estimating the time until the attack in EVS. Generative AI is a sub-branch of machine learning that stands out with its ability to generate data. It is used primarily in limited data sets to enrich data and create more diverse samples. This study presents an academic perspective on using generative artificial intelligence methods. Generative AI models can create new and diverse data samples from existing data sets. This is critical for increasing the model’s generalization ability and reducing the overfitting problem, especially when the data set is small or unbalanced. Generative AI models predict future data points in forecasting time series data. The proposed model architectıure presented in Fig. 7.

Integrating Generative Adversarial Networks (GANs) with a wide range of deep learning architectures offers a transformative approach to enhancing predictive modeling for cyber threats. This innovative collaboration takes advantage of the GAN’s exceptional ability to generate synthetic data while leveraging the distinctive strengths of various architectures tailored for specific types of data processing. This integration not only improves model robustness but also increases the accuracy of threat predictions.

This proposed model compellingly explores the capabilities of various Generative Adversarial Network (GAN) hybrid models in accurately predicting time series data, specifically focusing on Time_to_Next_Flag. It highlights the significant impact of various hybrid approaches in enhancing GAN performance for time-varying data. This presents a compelling case for their adoption in real-world applications, where timely defense strategies are essential. In the training Phase, the GAN Generator learns the distribution of cyber-attack patterns and generates realistic attack samples. Meanwhile, the GAN Discriminator differentiates between real and synthetic attack data. The chosen deep learning model (e.g., LSTM or GRU) processes both real and synthetic time-series data to predict the timing of attacks. To evaluate the model’s performance effectively, we utilize key metrics: Mean Absolute Error (MAE), Mean Squared Error (MSE), Root Mean Square Error (RMSE), and R-squared (R²). To effectively convey the implementation details, we present the Pseudocode of the proposed model in Algorithm 1.

**Figure:**
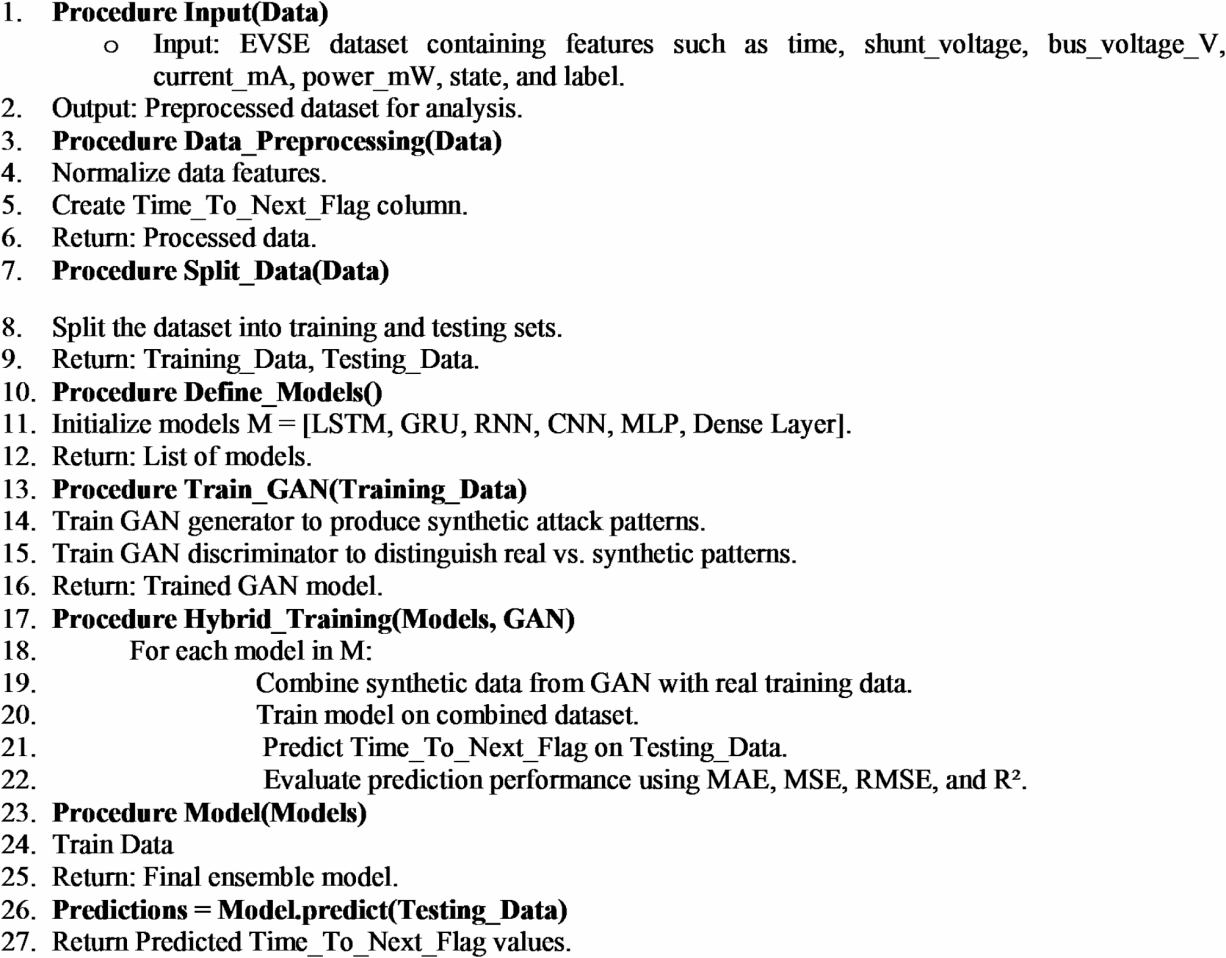
**Algorithm 1:** Proposed GAN-based hybrid model algorithmAlgorithm 1: Proposed GAN-based hybrid model algorithm

### Hybrid model created with the GAN and LSTM

GANs combined with LSTM networks are a powerful hybrid model, particularly in predicting sequential data with long-term dependencies. In cybersecurity contexts, such as predicting the time to the next attack on Electric Vehicle Supply Equipment (EVSE), the mentioned hybrid model leverages the ability of the GAN to produce synthetic attack patterns and the efficiency of the LSTM in learning from sequential data. The limited availability of attack data, especially for rare or new attacks, is among the significant difficulties in cybersecurity. GANs can create synthetic datasets representing possible cyberattacks and provide various scenarios to train predictive models. GANs produce realistic attack timelines and sequences fed into LSTM networks to better predict RUT by modeling future cyberattack patterns. Historical attack data are employed to train LSTM networks to recognize cyberattack patterns. By learning from these sequential patterns, the LSTM can predict the time of the next attack, ensuring that the system estimates the Remaining Useful Time (RUT) before a system failure or security breach. The hybrid GAN-LSTM model estimates the Remaining Useful Time (RUT) prior to an attack and, thus, offers an advanced solution for predicting cyberattacks in EVSE infrastructure. GANs present rich training data, simulating complex and rare attacks. On the other hand, LSTMs model temporal dependencies in cyberattacks effectively. This combination enhances the accuracy of attack predictions and ensures proactive defense mechanisms, increasing the resilience of EVSE systems to cyber threats.

The hybrid GAN-LSTM model has two main components: GAN and LSTM. GAN component: The generator in the GAN generates synthetic attack patterns, which are impossible to distinguish from accurate attack data. The discriminator assesses the generated data’s authenticity, allowing the model to improve the available training data by simulating different attack scenarios, enhancing the prediction model’s robustness. LSTM Component: LSTM, a recurrent neural network type, surpasses at capturing temporal dependencies in time-series data. In the hybrid model, the LSTM processes the generated and accurate data to predict the Time_to_Next_Flag—a critical metric in estimating the remaining time until a cyber attack.

#### Hybrid model created with the GAN and GRU

The purpose of this hybrid model, which combines the GAN and GRU, is to exploit the GAN’s generative capabilities with the sequential data processing and memory retention capabilities of GRUs. The GAN-GRU hybrid model first generates various cyberattack scenarios by utilizing the GAN and then trains the GRU to predict the likelihood and timing of future attacks based on real and synthetic data. This forms a more comprehensive and proactive defense mechanism. The GAN continuously generates novel attack scenarios, ensuring the GRU model is up-to-date with evolving cyber threats. As attackers adapt, the hybrid model can also adapt, which increases its effectiveness in forecasting future attacks, even under quickly changing conditions. The RUT strategy’s ability to predict the possible time of the next cyberattack is at its core. The hybrid model can make accurate time-to-attack predictions and allow system administrators to take preemptive action by analyzing the temporal dependencies captured by the GRU and the synthetic data generated by the GAN.

The hybrid use of GANs and GRUs for RUT strategies effectively predicts cyberattacks on EVSE systems. The approach generates synthetic attack data with GANs and leverages the time-series analysis capabilities of GRUs, enabling EVSE operators to anticipate attacks and employ proactive defense strategies.

#### Hybrid model created with the GAN and RNN

The present manuscript proposes a hybrid model integrating Generative Adversarial Networks (GANs) with Recurrent Neural Networks (RNNs) to enhance cyber attack detection in EVSE systems. This hybrid model’s architecture includes two main components: the generator and the discriminator (from the GAN) and the recurrent units (from the RNN). The generator produces synthetic cyberattack scenarios, and the discriminator evaluates them for authenticity. Afterward, the RNN processes sequential input data from real and synthetic sources to learn patterns and dependencies over time.

This hybrid model leverages the sequential nature of RNNs and the data generation capabilities of GANs, significantly increasing the accuracy and robustness of cyber attack predictions. The RNN continuously analyzes system behavior and compares it to previous attack patterns. Thus, the model updates the likelihood of an imminent cyberattack in real-time.

#### Hybrid model created with the GAN and CNN

The current section proposes a hybrid model, integrating Generative Adversarial Networks (GANs) with Convolutional Neural Networks (CNNs) in order to enhance the detection of cyber threats in Electric Vehicle Supply Equipment (EVSE). GANs generate synthetic cyberattack data in the GAN-CNN model, reflecting real-world attack vectors. These data are combined with real historical data from EVSE systems, which forms a comprehensive dataset to train the CNN. The CNN processes these data, detecting patterns and features that indicate abnormal system behavior, e.g., increased network traffic, unauthorized access attempts, or system anomalies. CNN analyzes the temporal patterns and features in the data to predict the time remaining before the next cyberattack.

The hybrid GAN-CNN model predicts cyberattacks in EVSE systems in a highly effective way by combining the GAN’s data generation capabilities with CNN’s powerful pattern recognition abilities. Due to this integration, the model can generate accurate Remaining Useful Time (RUT) predictions, which enables EVSE operators to take proactive measures to mitigate potential threats. The model’s robustness, adaptability, and reliability are improved by combining real and synthetic data, making it a valuable tool for protecting EV charging infrastructure against emerging cyber threats.

#### Hybrid model created with the GAN and MLP

The hybrid model, combining Generative Adversarial Networks (GANs) with the Multi-Layer Perceptron (MLP), leverages the data generation capabilities of GANs and the pattern recognition power of MLPs in order to increase the prediction of cyberattacks that target Electric Vehicle Supply Equipment (EVSE) and offers proactive defense mechanisms against potential attacks. In this hybrid approach, the MLP component maps complex input-output relationships. MLPs, which consist of multiple fully connected layers, effectively generalize data patterns. This makes them suitable for tasks that involve classification and prediction. GANs produce synthetic cyberattack data, which supplements the real-world data available for training. Therefore, the MLP becomes exposed to various attack scenarios, including edge cases that might be overlooked. After being trained on this comprehensive dataset, the MLP component can predict the Remaining Useful Time (RUT) before a cyberattack, which allows EVSE operators to take preventive action. The MLP effectively identifies patterns suggesting an impending attack, even in complex and nonlinear datasets. The hybrid model can be retrained since the GAN identifies or generates novel attack patterns, ensuring it remains current with evolving threats. This adaptability is crucial to maintaining effective cybersecurity defenses in a quickly changing threat landscape.

#### Hybrid model created with the GAN and dense layer

The present section explores the hybrid model, which integrates Generative Adversarial Networks (GANs) with Dense Layers, a critical neural network architecture often used in feedforward neural networks. GANs are incorporated to improve the predictive power of dense layers by generating synthetic data that enhances model training, particularly in scenarios with imbalanced or limited datasets.

A GAN framework has two main components: the generator and the discriminator. The generator must produce synthetic data that resemble real attack patterns. On the other hand, the discriminator assesses the authenticity of data, distinguishing between real and produced samples. Dense layers are known for their fully connected structure, in which each neuron is connected to every neuron in the preceding and subsequent layers. These layers are integrated into the generator and discriminator to deal with complex patterns in cyber threat data. The dense layer ensures that the model learns the complex relationships between different features associated with cyber attacks, enhancing its ability to produce data closely mirroring real-world scenarios. The discriminator, which also utilizes dense layers, receives real data from actual cyber attacks and synthetic data from the generator. Due to the dense connectivity, the discriminator evaluates the input, assigning a probability score to determine whether the data are real or produced. The dense layer architecture ensures that the discriminator captures slight differences between real and generated data, increasing its ability to distinguish between the two and enhancing the overall model’s robustness.

## Experimental results

Hence, these methods can create an effective cybersecurity strategy for EVS systems. We conducted this study in Python using the Google Colab Pro platform. We preferred the robust hardware infrastructure supported by NVIDIA’s A100 GPU, which has a capacity of 2 × 16G (32GB) RAM.

### Dataset

The data was collected in a laboratory environment established by the Canadian Institute for Cybersecurity (CIC)^26^. The dataset is structured around electrical measurements and cybersecurity events, capturing normal operations and attack patterns. The primary features consist of electrical parameters, including shunt voltage, bus voltage measured in volts (V), current measured in milliamperes (mA), and power measured in milliwatts (mW). The dataset includes two crucial target columns: a Label column identifying whether a particular instance represents an attack or normal behavior and a time_diff_to_next_attack column that measures the temporal distance to the next attack occurrence. Regarding attack patterns, the dataset specifically focuses on syn-flood attacks and denial-of-service attacks. This provides a focused lens for studying one specific type of cyber threat. The temporal aspects of the data are captured through time-based features. The primary time column records timestamps for each observation. This is complemented by the time_diff_to_next_attack feature, which provides crucial information about the temporal proximity of attack events. This combination of electrical measurements, attack labeling, and temporal features creates a comprehensive dataset for training and evaluating cybersecurity detection systems, particularly those focused on identifying syn-flood attacks in electrical infrastructure.

The comprehensive details of the dataset are clearly outlined in Table 2.

**Table 2:**
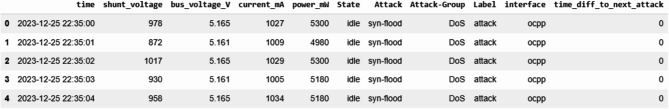
The details of the dataset^26^.

We took the dataset utilized in the current study from a real EV charging network on the campus of a large technology company. This included the CSV file for the EVSE-B’s power consumption under attack and benign conditions. The raw data consisted of 115,298 individual charging events, including the variables below: time, shunt_voltage, bus_voltage_V, current_mA, power_mW, State, Attack, Attack-Group, Label, and interface. The EVS data set consisted of data collected at one-minute intervals. Therefore, when the tag was created to calculate the time left for the attack, the problem was that all tags had the same value. This caused the applied models to fail.

We performed certain operations on the data set to solve the problem and create a more accurate labeling process.

First, upon examining the data set, we determined that more than 60 data were collected in the same minute in some cases, while less than 60 data were collected in other cases. This imbalance negatively affected model performance by causing inconsistencies in time series analysis. To solve the problem, we restructured the data set and limited the number of data in each minute to a maximum of 60 data.

We followed the steps below in the process of restructuring the data set:

Data Selection and Filtering: We selected 60 data points from the data collected within the same minute using the random selection method. The purpose was to form a more homogeneous data set by decreasing data density.

Updating the Time Column: We updated the time column, assuming that each data is collected at one-second intervals. We made this assumption to ensure data collection consistency and increase the effectiveness of time series analysis. Accordingly, the data was arranged between 0 and 59 s within a minute.

Label Creation: Based on the newly edited data set, we recalculated the labels representing the time remaining to the next attack for each data. This prevented tags from having the same value as before and ensured that more diversified and meaningful tags were created.

Afterward, we added the TIME_TO_NEXT_FLAG column; if there was an attack tag, we calculated the time remaining until the attack to be 0. If there was no attack tag, we calculated the difference in the time until the subsequent attack.

The novel data set obtained from these processes offers a more balanced time series structure. This structure ensures that more accurate and reliable labels are produced when calculating the time remaining for the attack. Thus, the performance of the models applied to the data set increased considerably, and more reliable results were yielded.

The data set consisted of 115,298 data. We obtained 116,298 data by adding 1000 more data with generative AI. We used 23,133 data as test data.

### Model performance metrics

To comprehensively and objectively assess the models’ performances, we selected the Mean Squared Error (MSE), Mean Absolute Error (MAE), Root Mean Square Error (RMSE), and R-squared (R²) as the models’ evaluation indicators.

- The MAE refers to a metric that measures the average magnitude of the errors between actual and predicted values without considering the error’s direction. It computes the absolute difference between the actual and predicted value for each observation and then averages these differences.The MAE is calculated as in Eq. (20):

20 $$MAE=\frac{1}{n}\mathop \sum \limits_{{i=1}}^{n} \left| {{y_i} - {{\hat {y}}_i}} \right|$$

- The MSE measures the average squared difference between the predicted and actual values. It is commonly utilized to assess regression models’ performance. The MSE is calculated as in Eq. (21):

21 $$MSE=\frac{1}{n}\mathop \sum \limits_{{i=1}}^{n} {\left( {{y_i} - {{\hat {y}}_i}} \right)^2}$$

- The RMSE is capable of visually expressing the model’s error. Nevertheless, outliers substantially impact it and must be assessed comprehensively with other indicators. The RMSE is calculated as in Eq. (22):

22 $$RMSE=\sqrt {\frac{1}{n}\mathop \sum \limits_{{i=1}}^{n} {{\left( {{y_i} - {{\hat {y}}_i}} \right)}^2}}$$

- The R² score, also called the coefficient of determination, represents a statistical measure indicating how well the model explains the variance in the dependent variable. The R² is calculated as in Eq. (23):

23 $${R^2}=1 - \frac{{\mathop \sum \nolimits_{{i=1}}^{n} {{\left( {{y_i} - {{\hat {y}}_i}} \right)}^2}}}{{\mathop \sum \nolimits_{{i=1}}^{n} {{\left( {{y_i} - \bar {y}} \right)}^2}}}$$

Where ${y_i}$ denotes the actual *i*^th^ value, ${\hat {y}_i}$ represents the predicted *i*^th^ value, and $\bar {y}$ refers to the average of the actual values.

### Results and discussion

The developed model’s predictive performance in estimating Time_To_Next_Flag was assessed by comparing the predicted values to actual outcomes. The results highlight the model’s behavior in over- and under-estimation scenarios and its effectiveness in providing accurate time-to-event predictions.

When the model performance is assessed based on the warning system, how many of the estimated values calculated using the above formula can be caught before the failure time and work as a correct warning system is determined.

**Fig. 8:**
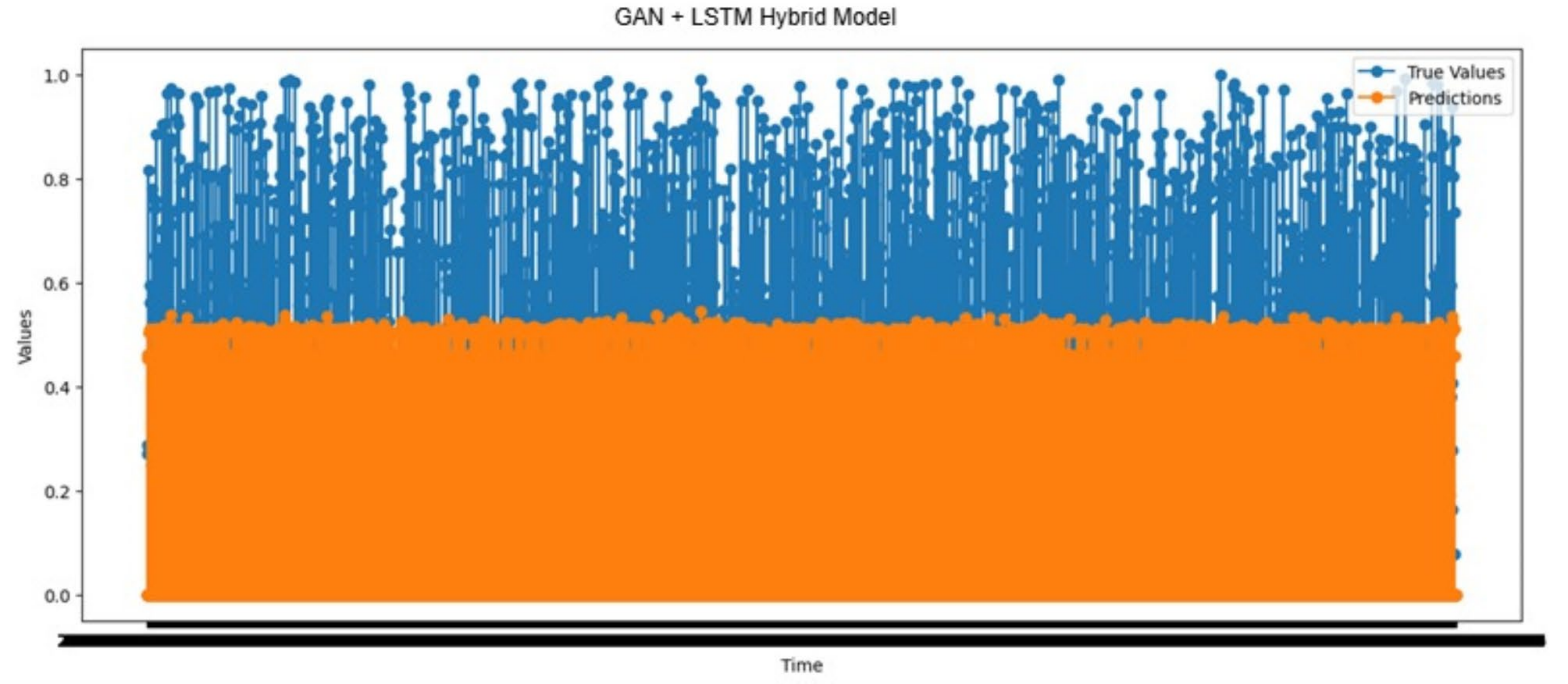
Actual values vs. predictions over time results of the GAN-LSTM hybrid model.

**Fig. 9:**
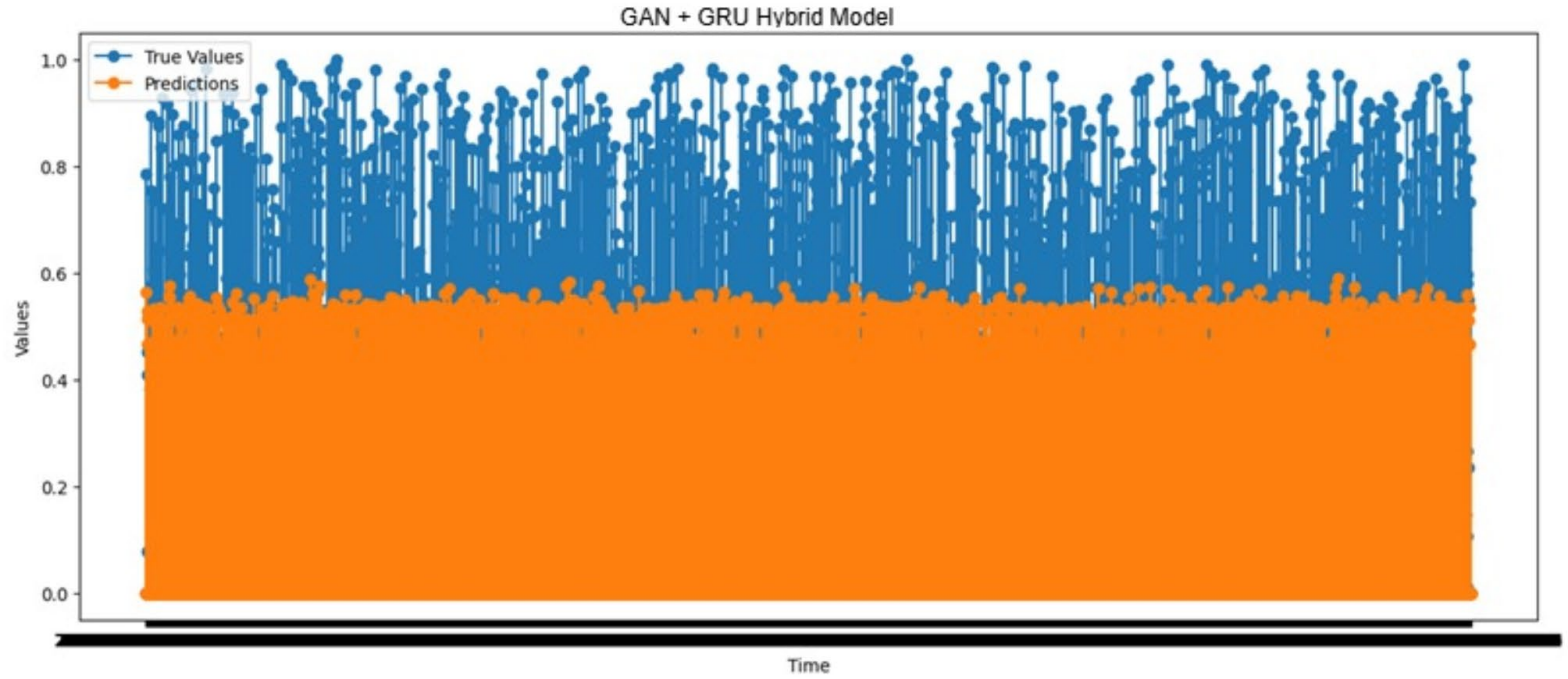
True values vs. predictions over time results of the GAN-GRU hybrid model.

**Fig. 10:**
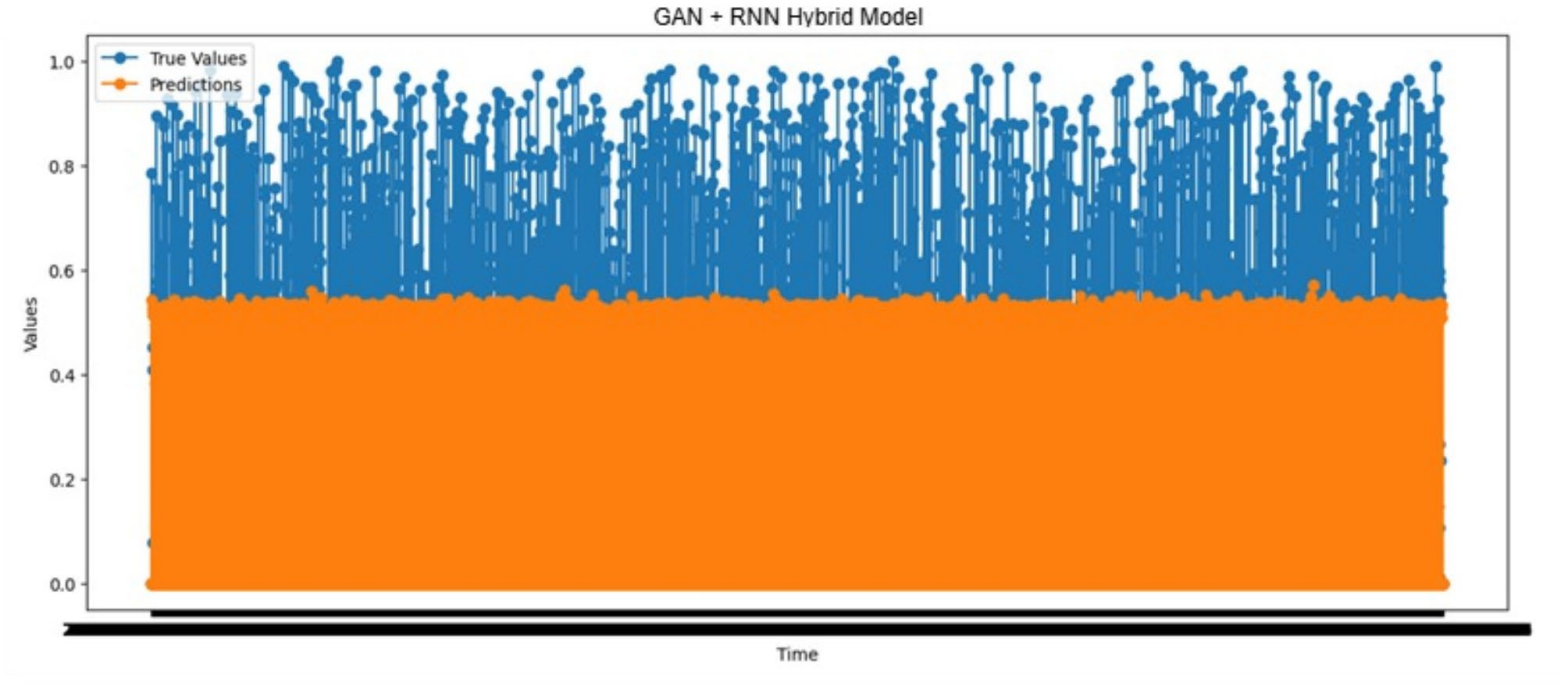
Actual values vs. predictions over time results of the GAN-RNN hybrid model.

**Fig. 11:**
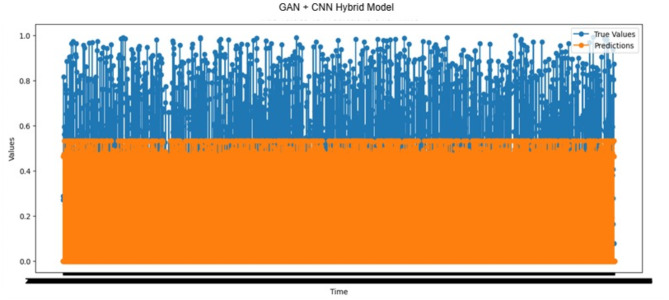
Actual values vs. predictions over time results of the GAN-CNN hybrid model.

**Fig. 12:**
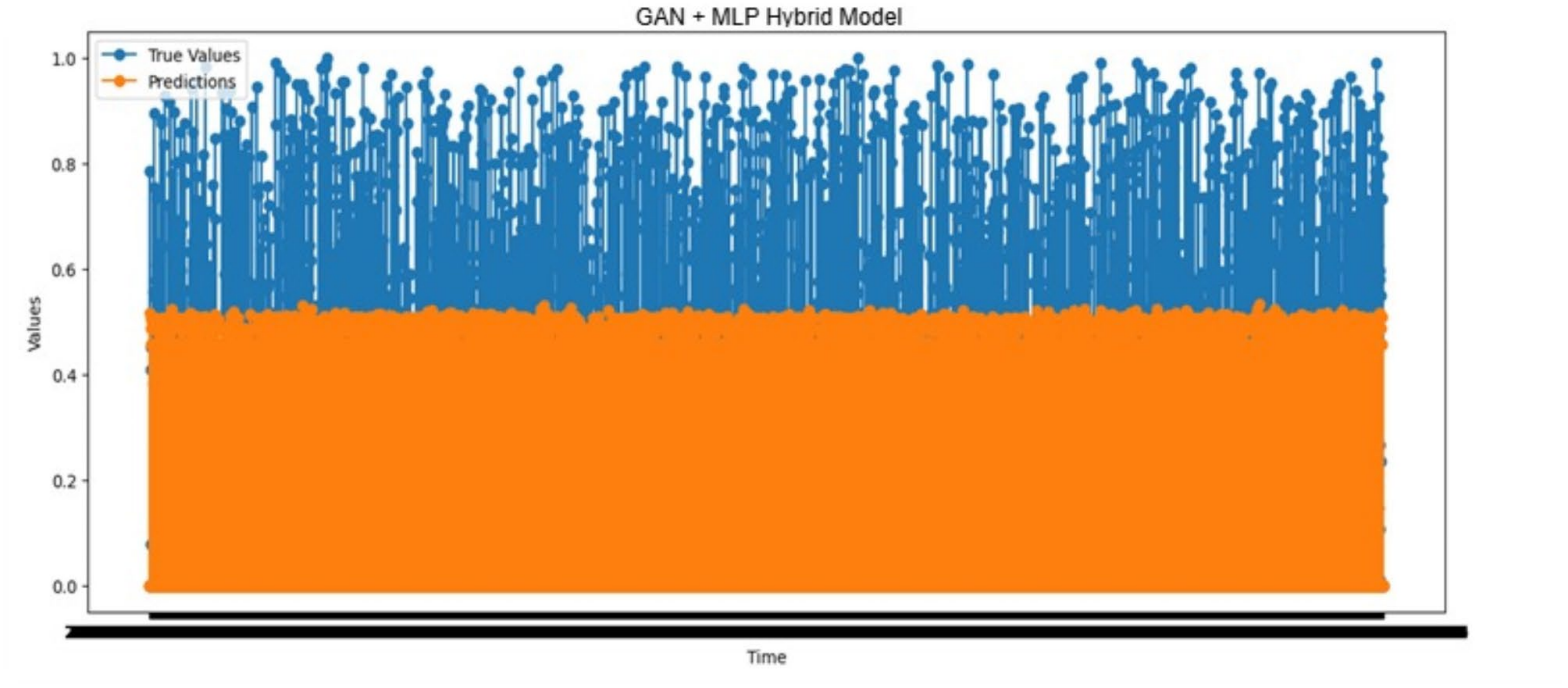
Actual values vs. predictions over time results of the GAN-MLP hybrid model.

**Fig. 13:**
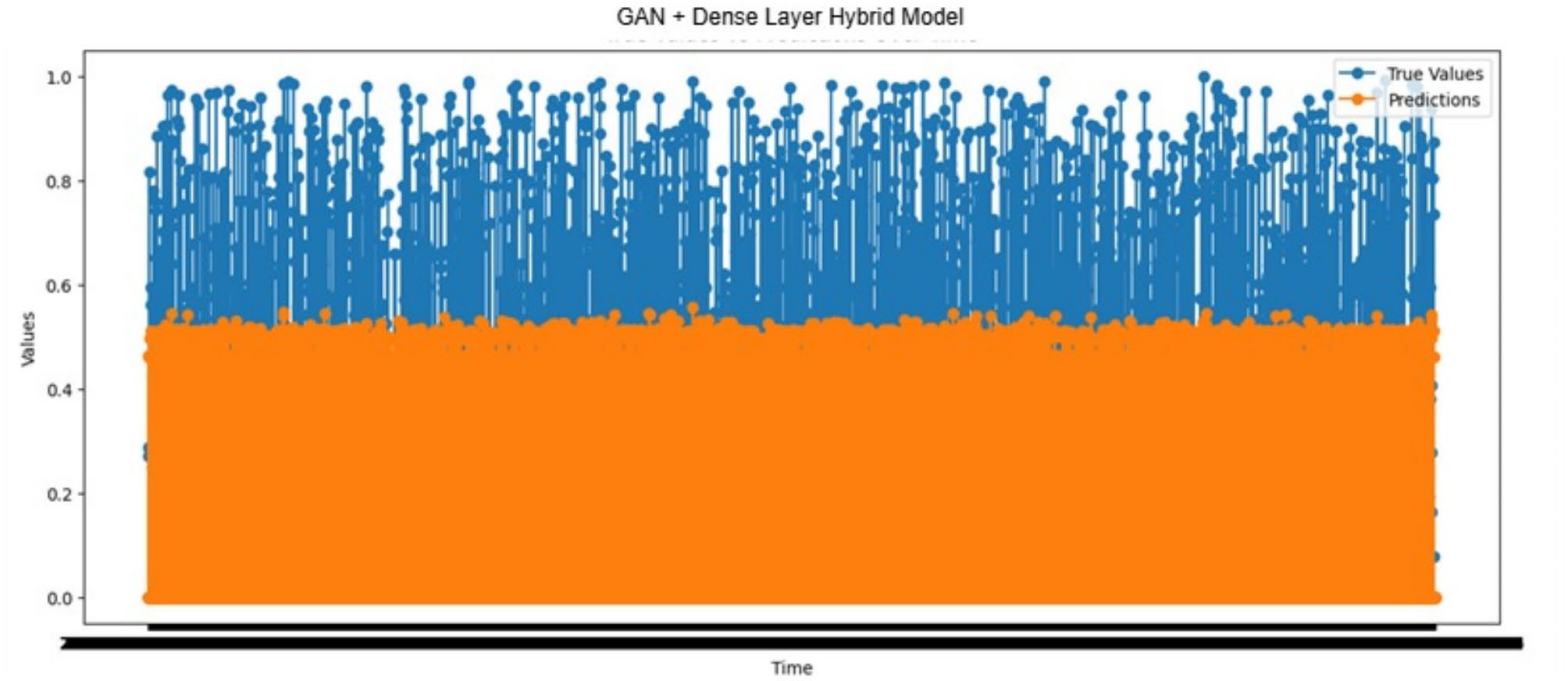
Actual values vs. predictions over time results of the GAN + Dense Layer hybrid model.

**Fig. 14:**
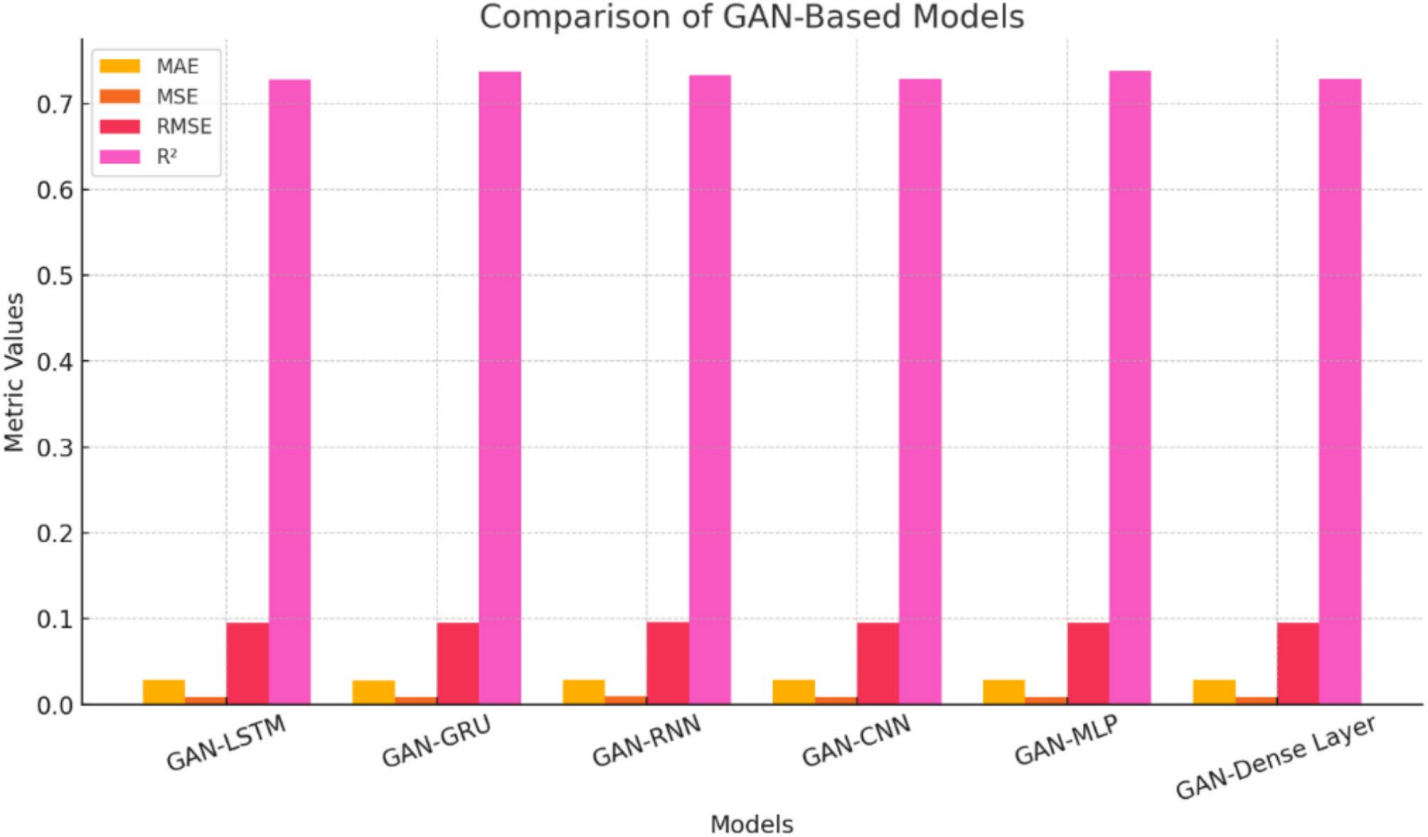
Actual values vs. predictions over time results of the GAN + Dense Layer hybrid model.

The numerical value under each figure (Figs. 8, 9, 10, 11, 12, 13 and 14) is the difference between the predicted and actual attack time and the predicted attack time being successful before the real attack time. Therefore, it should be considered a case, not an instance.

All models were meticulously trained for 50 epochs using the highly efficient Mean Squared Error (MSE) loss function. We split the dataset into 80% for training and 20% for testing, ensuring robust performance assessment with a batch size of 64. The Adam optimizer was employed with a learning rate 0.001, facilitating optimal convergence.

The experimental setup used a two-layer LSTM architecture, with 50 units in each layer. It employed Tanh and sigmoid activation functions. The model was optimized using the Adam optimizer, with a learning rate of 0.001 and a dropout rate of 0.2 to prevent overfitting. The GRU model is equally impressive, featuring two stacked GRU layers with 100 hidden units each. The first layer is configured to return sequences (Yes), providing valuable temporal information, while the second layer does not (No). The RNN model follows suit, consisting of two SimpleRNN layers of 100 hidden units each. It adheres to the same sequence return configuration as the GRU model, reinforcing its utility in time-series data. The CNN model harnesses the power of a Conv1D layer with 64 filters, followed by a Dense layer with 64 units, effectively eliminating the need for the “Return Sequences” parameter. The MLP model is expertly designed with three hidden layers of 128-64-32 sizes, rendering it fully connected and thus exempt from needing a “Return Sequences” parameter.

Finally, the Dense model is robustly structured with multiple layers: a Dense layer with 128 units followed by a LeakyReLU activation (0.01) and a Dropout layer (0.2), transitioning to another Dense layer with 64 units, which again employs LeakyReLU (0.01) and Dropout (0.2), culminating in a Dense layer with a single unit featuring a ReLU activation. Being a feedforward Dense model, it is not subject to the “Return Sequences” constraint, ensuring streamlined processing that reliably captures essential patterns in the data.

#### Prediction performance analysis using the GAN-LSTM hybrid model

Figure 8 illustrates the actual and expected outcomes of forecast Time_To_Next_Flag with the GAN-LSTM hybrid model. Out of the total cases, 1,423 cases exhibited predicted values greater than actual ones. This overestimation indicates that the model’s pre-warning mechanism may trigger prematurely, potentially leading to unnecessary system adjustments. On the contrary, the predicted value was lower than the actual one in 1,369 cases. This underestimation is crucial since it allows the system to issue timely preemptive warnings, improving its capability to prevent possible attacks. Reduced false negatives contribute directly to the system’s overall reliability and operational efficiency. Furthermore, in 20,441 cases, the remaining time until the attack was correctly estimated as zero, which indicates that the model accurately predicted imminent attacks. This shows the model’s ability to create an effective predictive mechanism to identify time-sensitive security breaches.

#### Prediction performance analysis using the GAN-GRU hybrid model

The actual and predicted outcomes of forecast Time_To_Next_Flag with the GAN-GRU hybrid model are shown in Fig. 9. Out of the total predictions, 1,467 cases had higher predicted values than the actual ones. This overestimation indicates a potential problem with pre-warning signals since early predictions may cause unnecessary system adjustments, which can disrupt the system’s stability. On the other hand, the predicted value was lower than the actual one in 1,382 cases. This underestimation is critical to the system’s reliability since it allows for the earlier detection of impending attacks. Reduced false negatives increase the system’s efficiency and improve its reliability in preventing potential threats. Additionally, in 20,441 cases, the time to the attack was correctly estimated as zero, indicating that the model effectively identified imminent attacks and provided accurate predictions. This confirms the ability of the model to establish an effective prediction mechanism for identifying system vulnerabilities.

#### Prediction performance analysis using the GAN-RNN hybrid model

Figure 10 illustrates the actual and predicted outcomes of forecasting Time_To_Next_Flag with the GAN-RNN hybrid model. In 1,552 cases, the predicted value was higher than the actual one, suggesting that the model triggered pre-warnings prematurely. While this may be considered a precautionary measure, frequent over-prediction can cause inefficiencies and unnecessary responses within the system. Excessive false positives can destabilize the system, emphasizing the need for the model to minimize such occurrences for enhanced system health and stability.

On the other hand, the predicted value was lower than the actual one in 1,297 cases. In contrast to over-prediction, under-prediction is crucial to decrease the likelihood of missed attacks by allowing preemptive actions. This lower number of false negatives shows that the system effectively identifies threats with sufficient warning, thereby increasing the overall reliability and efficiency of the prediction mechanism. A model with fewer false negatives is critical in robust defense systems since it ensures timely warnings without neglecting potential risks. Moreover, in 20,441 cases, the time remaining to the next attack was estimated as 0. The prediction was accurate in these cases. The result above indicates that the model can detect attacks when they are imminent and correctly predict the remaining time of the attack. Such precision in real-time forecasting is critical for system defense since it ensures that protective measures are executed timely, demonstrating the model’s potential as a powerful and reliable prediction tool. These observations demonstrate that the model accurately forecasts imminent attacks and effectively balances false positives and negatives. This performance, especially in reducing false negatives, enhances the model’s reliability, making it a critical component for any system that requires real-time prediction and defense mechanisms.

#### Prediction performance analysis using the GAN-CNN hybrid model

The actual and predicted outcomes of forecast Time_To_Next_Flag with the GAN-CNN hybrid model are given in Fig. 11. The predicted value exceeded the actual one in 1,434 cases, indicating that pre-warnings may not form a robust system structure based on these predictions. Conversely, the predicted value was lower than the actual one for 1,385 samples. This scenario, which includes fewer false negatives, can prevent potential attacks by allowing for advanced warnings, improving the efficiency and reliability of the system. Additionally, the remaining time to an attack was correctly estimated as zero in 20,441 cases, which showed the ability of the system to identify attacks and thus establish an accurate prediction mechanism.

#### Prediction performance analysis using the GAN-MLP hybrid model

The actual and predicted outcomes of forecast Time_To_Next_Flag with the GAN-MLP hybrid model are shown in Fig. 12. The predicted value exceeded the actual one in 1,418 cases, suggesting that pre-warnings based on such predictions may not establish a stable structure for the system. On the contrary, the predicted value was lower in comparison with the actual one in 1,431 cases, indicating the potential for preempting potential attacks through warning. Decreased false negatives increase the efficiency and reliability of the system by enabling more accurate attack forecasts. The remaining time until an attack was correctly predicted as zero in 20,441 cases. This demonstrates that the system effectively identifies attack scenarios and provides a robust attack prediction mechanism.

#### Prediction performance analysis using the GAN-dense layer hybrid model

The actual and expected outcomes of forecast Time_To_Next_Flag with the GAN-Dense Layer hybrid model are displayed in Fig. 13. The predicted values were more significant than those in 1,444 cases. Such overestimations suggest that issuing pre-warnings based on these predictions may not contribute to a healthy operational structure for the system. On the other hand, the predicted values were smaller than the actual ones in 1,375 samples. This scenario can preemptively prevent attacks since it allows for warnings, increasing the system’s efficiency and reliability through reduced false negatives. Moreover, the remaining time until an attack was accurately estimated as zero in 20,441 cases, demonstrating the system’s capability to identify attack scenarios accurately and confirming the effectiveness of the prediction mechanism employed.

The results illustrated in Fig. 14 indicate that all models achieve commendable R² values, ranging from approximately 0.70 to 0.75, demonstrating their overall efficacy. Notably, GAN-LSTM and GAN-GRU exhibit slightly higher R² values, around 0.73, whereas GAN-Dense Layer, while still respectable, has the lowest value at approximately 0.72. A review of the error metrics reveals consistently low figures across all models: the Mean Absolute Error (MAE) falls between 0.03 and 0.04, the Mean Squared Error (MSE) ranges from 0.01 to 0.02, and the Root Mean Squared Error (RMSE) shows similar values between 0.09 and 0.10. The sequential models, which include GAN-LSTM, GAN-GRU, and GAN-RNN, demonstrate marginally superior performance in terms of R² and slightly lower error metrics. Among these, GAN-LSTM stands out as the top performer. GAN-CNN exhibits results comparable to those of the sequential models, while GAN-MLP shows a slight decrease in performance, and the GAN-Dense Layer demonstrates the lowest overall effectiveness. Despite minor variations in performance, all models achieve impressive results with R² values exceeding 0.70. The error metrics remain consistently low across the different architectures. Notably, sequential models, particularly GAN-LSTM, show a slight advantage in accuracy. These findings strongly suggest that, while variations exist among the models, all tested GAN architectures represent robust and reliable options tailored to specific applications, making them valuable choices for enhancing predictive performance.

Table 3 summarizes the performance of diverse models integrating Generative Adversarial Networks (GANs) with various DL architectures. MAE, Mean MSE, RMSE, and R-squared (R²) are among the metrics used for evaluation. The MAE values across the models varied between 0.0281 and 0.0291. The GAN-GRU model exhibited the lowest MAE at 0.0281, indicating the highest accuracy in its predictions. On the contrary, the GAN-RNN model had the highest MAE at 0.0291, suggesting slightly lower accuracy than the other models. All models had relatively low MAE values, demonstrating that their predictions were generally close to the actual values. The MSE values varied between 0.0089 and 0.0092, with the GAN-CNN model performing the best at 0.0089. The GAN-RNN model had the highest MSE at 0.0092, indicating its tendency to produce more significant errors. All models generally maintained low MSE values, which implied that significant prediction errors were infrequent across all architectures. The RMSE values ranged from 0.0948 to 0.0958. The GAN-CNN model again outperformed the others with the lowest RMSE at 0.0948, reflecting a consistent and low prediction error rate. The GAN-RNN model had the highest RMSE at 0.0958, which showed slightly less consistency and accuracy in its predictions than the others. The R² values indicate the proportion of variance explained by the models, ranging from 0.7280 to 0.7379. The GAN-MLP model had the highest R² value of 0.7379, suggesting that it explained about 73.79% of the variance in the data, which is a strong performance. The GAN-LSTM model had the lowest R² value of 0.7280. Nevertheless, it still suggested a reasonable explanatory power.

**Table 3:** The performance of the GAN-based hybrid DL models.

	GAN-LSTM	GAN-GRU	GAN-RNN	GAN-CNN	GAN-MLP	GAN-Dense Layer
MAE	0.0285	0.0281	0.0291	0.0285	0.0288	0.0285
MSE	0.0090	0.0090	0.0092	0.0089	0.0090	0.0090
RMSE	0.0950	0.0950	0.0958	0.0948	0.0949	0.0949
R²	0.7280	0.7377	0.7334	0.729	0.7379	0.7288

Despite the promising results, several limitations remain. First, the models rely on historical attack data, which may not effectively capture emerging and adaptive cyber threats. The generative capabilities of GANs help mitigate this limitation, but further refinement is needed to ensure the models remain robust against novel attack strategies. Second, the high computational cost of training hybrid deep learning models can hinder real-time deployment, particularly for large-scale EVSE networks. Third, the potential for false positives and negatives, while minimized, still requires further optimization to enhance detection efficiency and prevent unnecessary system alerts.

Future research should focus on the following directions:

- Enhancing Generalization and Robustness: Exploring advanced adversarial training techniques to improve model resilience against evolving cyber threats. Introducing reinforcement learning-based adaptation mechanisms can also enable models to self-improve based on real-time threat scenarios.
- Real-Time Implementation: Optimizing computational efficiency to enable real-time deployment of GAN-based hybrid models in EVSE systems. Techniques such as model pruning, quantization, and edge computing can enhance performance.
- Integration with Blockchain and Secure Communication Protocols: Machine learning-based attack detection combined with blockchain technology creates a secure and tamper-proof EVSE network. Implementing decentralized security protocols can further reduce vulnerabilities.
- Transfer Learning and Few-Shot Learning: Investigating methods that allow models to learn from limited attack instances and generalize across different EVSE infrastructures without requiring extensive retraining.
- Multi-Modal Cybersecurity Approaches: Combining deep learning models with traditional cybersecurity techniques, such as signature-based detection and heuristic analysis, creates a more comprehensive defense system.

Ethical and Regulatory Considerations: Examining the ethical implications and regulatory requirements for deploying AI-driven cybersecurity systems in critical infrastructure like EVSE. Ensuring compliance with global cybersecurity standards is crucial for widespread adoption.

By addressing these challenges, future studies can further enhance the security of electric vehicle charging infrastructure, ensuring a proactive defense against cyber threats while improving the reliability and resilience of EVSE systems.

## Conclusion

The effectiveness of hybrid models integrating GANs with various deep learning architectures, such as LSTM, GRU, RNN, CNN, MLP, and dense layers, was highlighted in the present work. The proposed GAN-based models were specifically tailored to enhance the predictive performance for estimating the time until the next cyber attack in EVSE systems. Among the different architectures we assessed, the GAN combined with GRU yielded the best results, achieving the lowest MAE and effectively minimizing false positives and false negatives. This demonstrates a robust performance in detecting imminent threats while maintaining the system’s stability. The models could make more reliable predictions by restructuring the dataset and ensuring a balanced representation of attack scenarios. In conclusion, integrating GANs with advanced deep learning techniques enhances predictive modeling capabilities in cybersecurity. It paves the way for developing more resilient systems to safeguard against emerging threats effectively. Future research should further optimize these hybrid models and explore additional applications within other domains susceptible to cyber threats.

## Data Availability

Data is available from the Laith Abualigah upon reasonable request. The datasets generated and/or analysed during the current study are available in https://www.kaggle.com/datasets/geoffnel/evs-one-electric-vehicle-dataset.
